# Host Retromer Protein Sorting Nexin 2 Interacts with Human Respiratory Syncytial Virus Structural Proteins and is Required for Efficient Viral Production

**DOI:** 10.1128/mBio.01869-20

**Published:** 2020-09-29

**Authors:** Ricardo S. Cardoso, Lucas Alves Tavares, Bruna Lais S. Jesus, Miria F. Criado, Andreia Nogueira de Carvalho, Juliano de Paula Souza, Sukhmani Bedi, Marcos Michel de Souza, Maria Lucia Silva, Guilherme Pauperio Lanfredi, Brenda Cristina Vitti, Orlando Bonito Scudero, Vitor Marcel Faça, Akira Ono, Armando M. Ventura, Luis Lamberti P. daSilva, Eurico Arruda

**Affiliations:** a Department of Cell and Molecular Biology, University of São Paulo School of Medicine, Ribeirao Preto, Brazil; b Department of Microbiology, Institute of Biomedical Sciences, University of São Paulo, São Paulo, Brazil; c Department of Biochemistry, University of São Paulo School of Medicine, Ribeirao Preto, Brazil; d Department of Microbiology and Immunology, University Michigan Medical School, Ann Arbor, Michigan, USA; Columbia University Medical College

**Keywords:** human respiratory syncytial virus, paramyxovirus, protein trafficking, virus-host interactions

## Abstract

Human respiratory syncytial virus (HRSV) envelope glycoproteins traffic to assembly sites through the secretory pathway, while nonglycosylated proteins M and N are present in HRSV inclusion bodies but must reach the plasma membrane, where HRSV assembly happens. Little is known about how nonglycosylated HRSV proteins reach assembly sites. Here, we show that HRSV M and N proteins partially colocalize with the Golgi marker giantin, and the glycosylated F and nonglycosylated N proteins are closely located in the trans-Golgi, suggesting their interaction in that compartment. Brefeldin A compromised the trafficking of HRSV F and N proteins and inclusion body sizes, indicating that the Golgi is important for both glycosylated and nonglycosylated HRSV protein traffic. HRSV N and M proteins colocalized and interacted with sorting nexin 2 (SNX2), a retromer component that shapes endosomes in tubular structures. Glycosylated F and nonglycosylated N HRSV proteins are detected in SNX2-laden aggregates with intracellular filaments projecting from their outer surfaces, and VPS26, another retromer component, was also found in inclusion bodies and filament-shaped structures. Similar to SNX2, TGN46 also colocalized with HRSV M and N proteins in filamentous structures at the plasma membrane. Cell fractionation showed enrichment of SNX2 in fractions containing HRSV M and N proteins. Silencing of SNX1 and 2 was associated with reduction in viral proteins, HRSV inclusion body size, syncytium formation, and progeny production. The results indicate that HRSV structural proteins M and N are in the secretory pathway, and SNX2 plays an important role in the traffic of HRSV structural proteins toward assembly sites.

## Abstract (precis)

The present study contributes new knowledge to understand HRSV assembly by providing evidence that nonglycosylated structural proteins M and N interact with elements of the secretory pathway, shedding light on their intracellular traffic. To the best of our knowledge, the present contribution is important given the scarcity of studies about the traffic of HRSV nonglycosylated proteins, especially by pointing to the involvement of SNX2, a retromer component, in the HRSV assembly process.

## INTRODUCTION

Human respiratory syncytial virus (HRSV) is the most relevant viral cause of respiratory infection in children, with extensive morbidity and nearly 200,000 deaths every year (1). HRSV belongs to the genus *Orthopneumovirus* of the family *Pneumoviridae* and has a negative-strand RNA genome that encodes the following 11 proteins: the nonstructural proteins 1 and 2 (NS1 and NS2), nucleoprotein (N), phosphoprotein (P), matrix protein (M), small hydrophobic (SH), major glycoprotein (G), fusion (F) glycoprotein, proteins M2-1 and -2, and the polymerase (L) (2). To enter host cells, the HRSV surface glycoproteins bind to several possible cell surface receptors (3–5), triggering fusion of the virus envelope with the cell membrane, mediated by the cleaved viral protein F (6). HRSV glycoproteins are translated in association with the endoplasmic reticulum (ER), glycosylated in the Golgi apparatus, and then targeted to virus assembly/budding sites through the secretory pathway (2). In polarized epithelial cells, HRSV assembly takes place at the apical plasma membrane, taking advantage of the apical endosome recycling pathway with the incorporation of caveolin-1 in virus particles (7–9). HRSV F glycoprotein, in particular, traffics to the apical membrane following the secretory pathway, and its cytoplasmic tail is important for this addressing (10). In this context, RAB11-FIP2 protein was shown to be critical for virus egress, since HRSV is not dependent on the Vps-4, an element of the ESCRT machinery, for its replication (11). Remarkably, although viral glycoproteins are important components of the virus structure, they are not essential for targeting the virus assembly/budding processes toward the apical surface of polarized cells (12, 13).

Proteins N and M have multiple functions during HRSV replication. The N protein directly interacts with the viral RNA, participates in the formation of virus-induced inclusion bodies, and has been shown to impair immunological synapses on the cell surface (14), whereas the M protein participates in virus assembly and budding on the cell surface and interacts with different host cell proteins to play several other functions in the virus replication (15–17). It was recently shown by superresolution imaging that the inclusion bodies are compartmentalized viral factories with specific regions where viral mRNA transcription happens involving N, P, L, M2-1, and viral genome (18). In contrast to the HRSV envelope glycoproteins whose traffic through the Golgi apparatus has been well studied, very little is known about how the nonglycosylated M and N structural proteins are conveyed to virus assembly sites, which prompted the present studies.

## RESULTS

### HRSV F, M, and N proteins colocalize at the Golgi.

**FIG 1:**
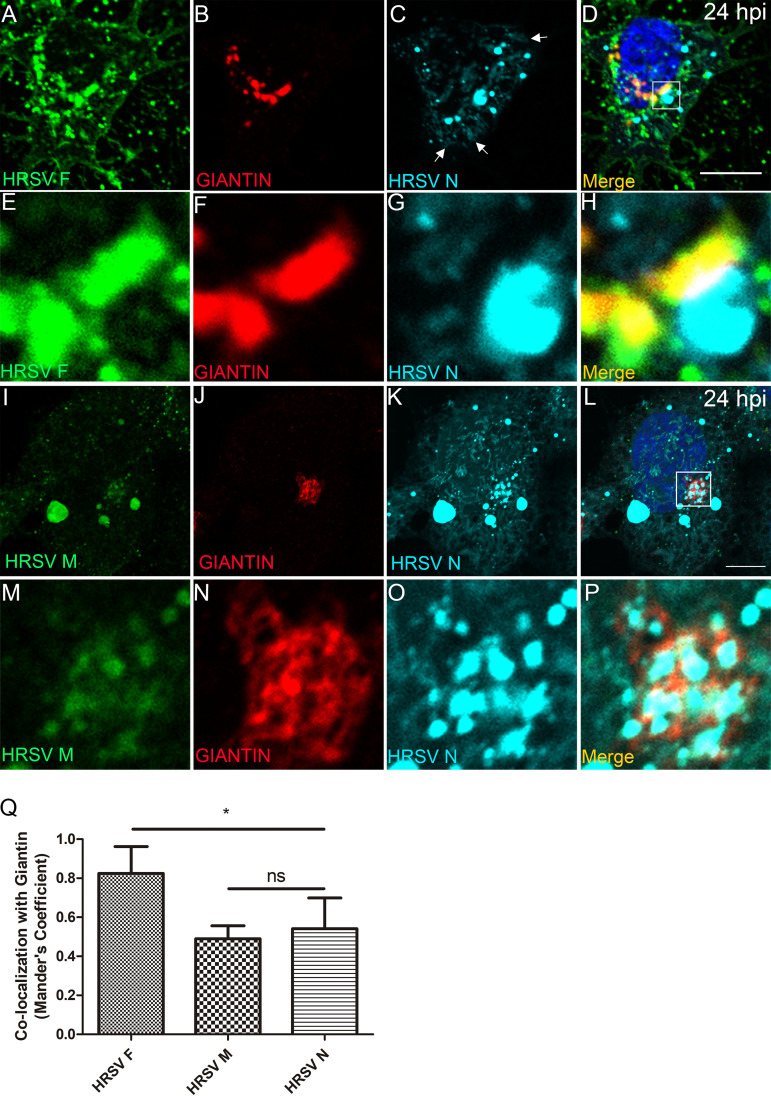
HRSV M and N proteins colocalize with the cis and medial Golgi marker giantin. (A to D) Colocalization of glycosylated HRSV F (A), giantin (B), and HRSV nonglycosylated N protein (C). (E to H) Higher magnification of panels A, B, C, and D, respectively. (I to L) Colocalization of HRSV M (I), giantin (J), and HRSV N (K). (M to P) Higher magnification of panels I, J, K, and L, respectively. (Q) Mander’s coefficient of colocalization of HRSV F (used as a positive control of colocalization with the Golgi), M, and N proteins with giantin. This set of immunofluorescence images is a single plane, representative of at least three independent experiments. The Mander’s coefficient was calculated from Z-stack images of at least five cells from three independent experiments. The *P* value was determined using analysis of variance (ANOVA) one-way Tukey’s multiple-comparison test. *, *P* < 0.05; **, *P* < 0.01; ***, *P* < 0.001; ns, nonsignificant. Images were taken at 24 hpi infection with a Zeiss 780 confocal microscope. Magnification, ×63. All scale bars = 10 μm.

Although it is well established that HRSV assembles at the plasma membrane (2), it is possible that the structural proteins F, M, and N reach a common intracellular site. To investigate this possibility, we studied their spatial relationship with markers of the secretory and endocytic pathways in infected cells. The HRSV F glycoprotein is synthesized in the ER, and it is known to traverse the Golgi stacks (19, 20). In fact, at 24 hours postinfection (hpi), the F protein largely colocalized with giantin, a marker of cis and medial Golgi cisternae at the juxtanuclear region (Fig. 1A to H and Q), and it is also present at the cell periphery. N protein labeling was also detected at the cell periphery in filament-shaped structures (arrows in Fig. 1C) and concentrated at large vesicular structures, suggestive of inclusion bodies, which were often seen in close proximity to giantin, and F protein labeling (Fig. 1A to P and Q). In addition to large cytosolic vesicles, M protein labeling was also detected throughout cytoplasm and at the juxtanuclear region, where it partially overlapped with giantin (Fig. 1I to Q and Fig. S1G to J). Therefore, this set of data indicates that there is partial overlap of the HRSV F, M, and N proteins at the cis and medial Golgi.

10.1128/mBio.01869-20.1 FIG S1 HRSV inclusion bodies are surrounded by the Golgi marker giantin. (A to C) Panel C shows colocalization between HRSV N (A) and giantin (B). (D, E, and F) Higher magnification of panels A, B, and C, respectively. Panel F shows HRSV inclusion bodies surrounded by giantin. (G to J) Panel G shows colocalization between HRSV N (H), giantin (I), and HRSM M (J), with arrowheads pointing to places where the HRSV proteins colocalize with giantin, and the arrow pointing to a cell where there is no colocalization between HRSV proteins and the Golgi at this focal plane. Approximately 73% of the infected cells containing inclusion bodies showed this pattern of localization to some extent. All images were taken at 24 hpi with a Zeiss 780 confocal microscope and are a representation of at least three different experiments. Figures shown are a single focal plane from Z-stack imaging. Magnification, ×63. Scale bar = 10 μm. Download FIG S1, PDF file, 0.4 MB. Copyright © 2020 Cardoso et al. 2020 Cardoso et al. This content is distributed under the terms of the Creative Commons Attribution 4.0 International license.

**FIG 2:**
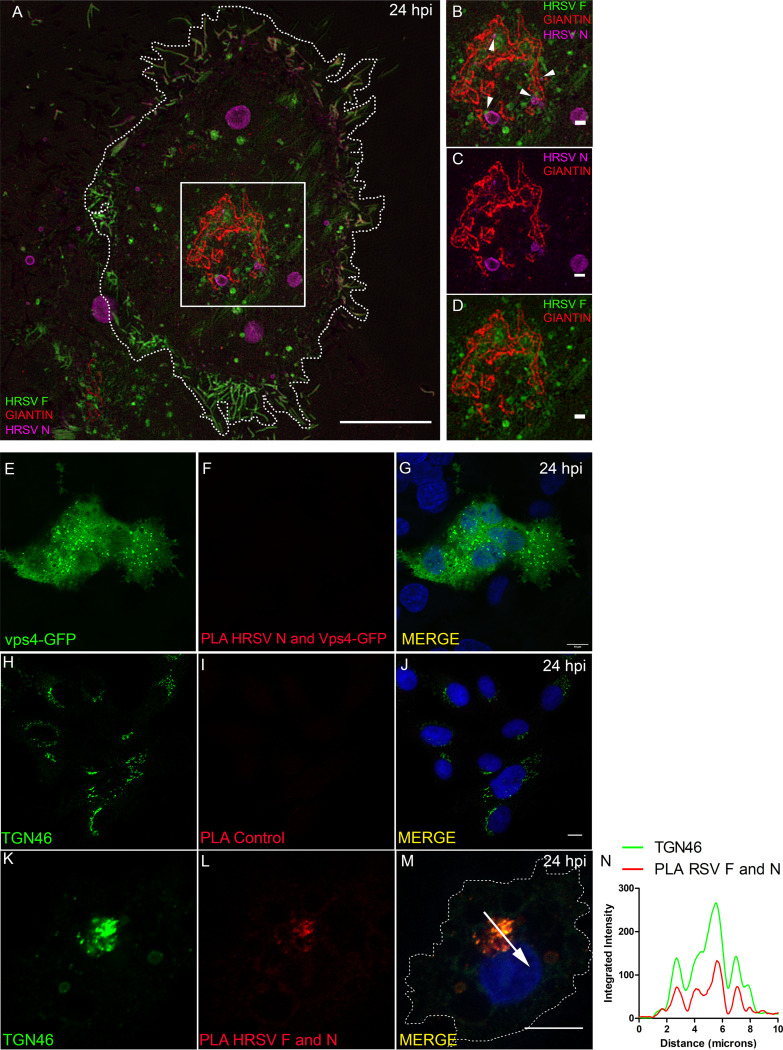
Superresolution in microscopy and PLA confirm that HRSV N and F proteins are in tight proximity. (A) Overview of an HRSV-infected cell at 24 hours postinfection displaying HRSV F in green, giantin in red, and HRSV N in magenta. (B) Higher magnification of panel A evidencing the tight proximity between HRSV F and N proteins at the Golgi (arrowheads). (C) Higher magnification of panel A showing the HRSV N staining the inclusion bodies, associated with Golgi membranes. (D) Higher magnification of panel A showing the HRSV F protein in green associated with the Golgi membranes in red. The cell nuclei staining was omitted. (E) HRSV infected and transfected with Vps4-GFP plasmid. (F) PLA for HRSV N and Vps4-GFP performed in HRSV-infected cells and transfected with Vps4-GFP plasmid. (G) A merge between panels E and F. (H) HRSV-infected cells stained for TGN46. (I) PLA performed in HRSV-infected cells without using the HRSV N primary antibody as a biological control of the experiment. (J) Merge between panels H and I. (K) TGN46 in HRSV-infected cells. (L) PLA for HRSV F and N proteins. (M) Merge of panels K and L. (N) Proximity/interaction of F and N occurs at the Golgi compartment; approximately 35% of the cells analyzed showed PLA positive staining as in panels L and M. The plot profile in panel N was performed following the arrow traced in panel M. Panels A to D represent a single plane from a Z-stack imaging from a Nikon N-SIM microscope (superresolution imaging), while E to M represent a single focal plane of two independent experiments taken with a Zeiss 780 confocal microscope. Magnification, ×63. All scale bars = 10 μm, except for panels B, C, and D, in which the scale bar = 1 μm.

This notion was confirmed by superresolution imaging showing tight proximity between HRSV F and N proteins in giantin-labeled Golgi compartments (Fig. 2A to D and Video S1). To test if F and N proteins interact with each other, proximity ligation assays (PLA) were performed (Fig. 2E to N). PLA signal for HRSV F and N proteins demonstrated that interaction occurs near the Golgi compartments, as shown by the partial overlap of PLA and TGN46 signals (Fig. 2K to N). It is noteworthy that TGN46 labeling was used instead of giantin to localize the Golgi, because PLA requires antibodies made in different species, in this case rabbit and mouse. Since the antibody for giantin was raised in rabbit, to overcome this limitation we used TGN46 antibody made in sheep. As negative control of PLA, cells were transfected with Vps4-GFP, and at 4 hours posttransfection, the cells were infected with HRSV. At 24 hpi the cells were fixed, and the PLA protocol for HRSV N and GFP was performed (Fig. 2E to G). Since it has been known that the HRSV assembly process is independent of Vps4 (11), Vps4 becomes a good PLA negative control. As an additional PLA negative control, HRSV-infected cells were probed for PLA omitting one of the two primary antibodies (Fig. 2H to J). Taken together, these results suggest that HRSV F and N proteins may interact with each other in the Golgi before reaching the plasma membrane.

10.1128/mBio.01869-20.5 SUPPLEMENTARY VIDEO 1 Three-dimensional (3-D) video of the cropped area in Fig. 2A. 3-D reconstruction was performed with Fiji J software. The virtual Z sections represent 17 images. HRSV F is seen in green, giantin in red, and HRSV N in magenta. Download Video S1, AVI file, 0.4 MB. Copyright © 2020 Cardoso et al. 2020 Cardoso et al. This content is distributed under the terms of the Creative Commons Attribution 4.0 International license.

### HRSV N and M proteins partially colocalize with the trans-Golgi network marker TGN46.

**FIG 3:**
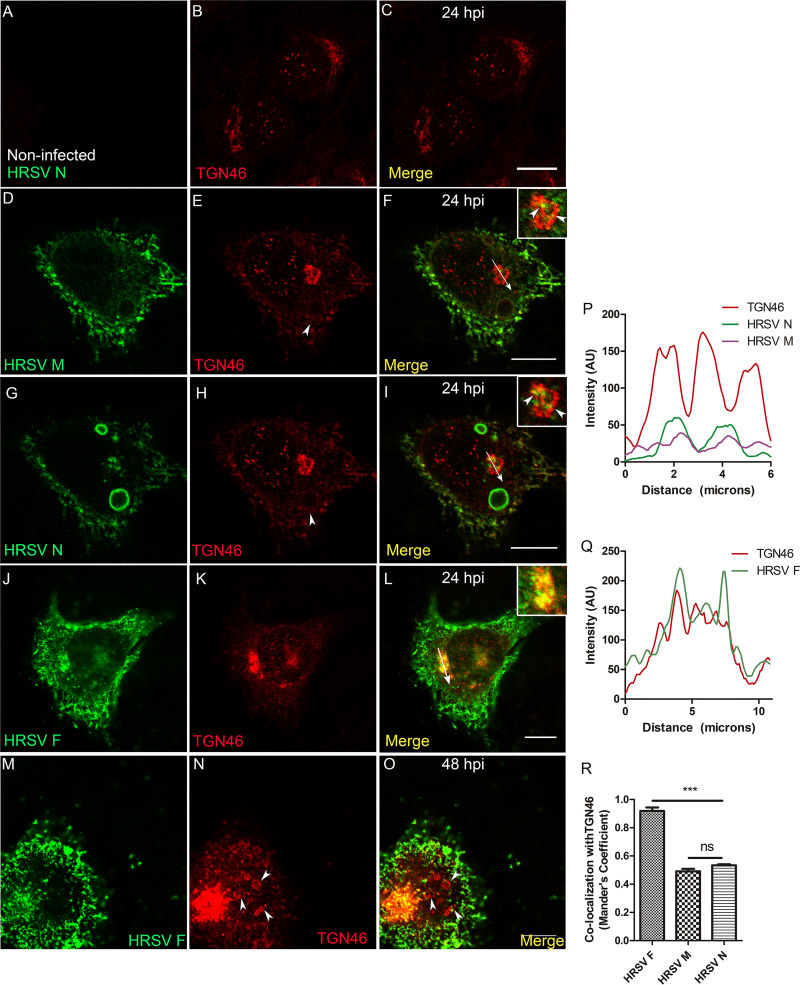
HRSV M and N proteins colocalize with the trans-Golgi network marker TGN46. (A to C) Negative control showing staining for TGN46 in noninfected cells. (D and E) Separate channels of HRSV M protein and TGN46. (F) Colocalization of HRSV M protein and TGN46, with arrowheads indicating the points of colocalization and the arrow where the plot profile was traced. (G and H) Separate channels of HRSV N protein and TGN46. (I) The same cell as in panel F, showing colocalization of HRSV N protein with TGN46, with an arrow indicating where the plot profile was traced and arrowheads indicating points of colocalization. (J and K) Separate channels of HRSV F protein and TGN46. (L) Colocalization of HRSV F protein and TGN46 at 24 hpi. (M and N) Separate channels of HRSV F protein and TGN46, with arrows pointing to the ring-shaped structures labeled for TGN46. (O) Colocalization of HRSV F and TGN46. (P) Plot profile of panels D to I. (Q) Plot profile corresponding to the arrow traced in panel L. (R) Mander’s colocalization between HRSV F, M, and N with TGN46 at 24 hpi. Panels A to O represent a single focal plane of at least three independent experiments taken with a Leica SP5 confocal microscope. Magnification, ×63. The colocalization *P* value was determined using ANOVA one-way Tukey’s multiple-comparison test. *, *P* < 0.05; **, *P* < 0.01; ***, *P* < 0.001; ns, nonsignificant. All the scale bars = 10 μm.

Next, we investigated whether HRSV N and M proteins reach the endosomal system and the plasma membrane via the trans-Golgi network (TGN). To this end, we labeled for TGN46, a transmembrane protein that cycles between early endosomes and the TGN, where it is mostly localized at steady state (21). As expected, TGN46 is concentrated in the juxtanuclear region in noninfected cells (Fig. 3A to C). In infected cells, TGN46 partially colocalized with HRSV N and M proteins that were apart from typical inclusion bodies (Fig. 3D to I and P). In addition, superresolution imaging demonstrated that it is possible to see HRSV N, which is not associated with inclusion bodies, appearing tightly associated with the TGN46 (Video S2). However, the colocalization of either HRSV M or N with TGN46 was significantly lower than that of HRSV F and TGN46 (Fig. 3J, L, P to R) at 24 hpi. This is in agreement with a previously published study (14) showing that HRSV N protein is localized in the TGN. However, our findings suggest that the colocalization of TGN46 with the HRSV M and N proteins is moderate. We conclude that the HRSV M and N proteins partially colocalized with TGN46. Interestingly, TGN46 also showed a distribution pattern at the cell periphery and surrounding structures that resemble HRSV inclusion bodies in ring-shaped structures that were apparent at 24 hpi (Fig. 3D to I, pointed by arrowheads in Fig. 3E and H). To rule out immunofluorescence bleeding from the channels coming from the HRSV inclusion bodies, cells were infected with HRSV, and at 48 hpi they were stained for TGN46. To demonstrate that these cells are actually infected, we stained them for HRSV F protein, instead of staining them for HRSV proteins that are components of inclusion bodies (Fig. 3M to O). It is possible to see that ring-shaped structures became more evident at 48 hpi (Fig. 3M to O) and that they are not a result of the fluorescence signal bleeding at the moment of the imaging acquisition. Because the TGN localization of TGN46 is maintained by efficient recycling from endosomes (22), these results may suggest that this retrieval pathway could be compromised in late infection.

10.1128/mBio.01869-20.6 SUPPLEMENTARY VIDEO 2 Three-dimensional video of the cropped area in Fig. 10T. 3-D reconstruction was performed with Fiji J software. The virtual Z sections represent 17 images. HRSV N is seen in green and TGN46 in red. Download Video S2, AVI file, 3.9 MB. Copyright © 2020 Cardoso et al. 2020 Cardoso et al. This content is distributed under the terms of the Creative Commons Attribution 4.0 International license.

### HRSV glycosylated and nonglycosylated proteins depend on the Golgi integrity to reach the cell surface.

**FIG 4:**
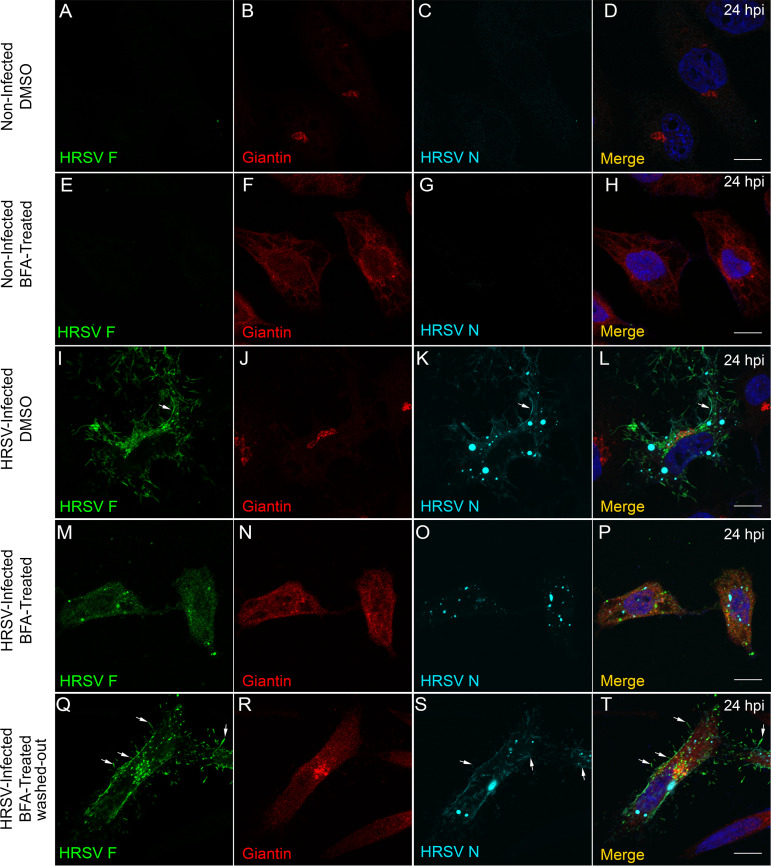
Treatment with brefeldin A affects the distribution of HRSV F and N proteins. (A) Negative control showing lack of staining for HRSV F in noninfected cells. (B) Intact Golgi upon treatment with DMSO used as vehicle for BFA. (C) Negative control showing staining for HRSV N in noninfected cells. (D) Merge of panels A, B, and C. (F) Effect of BFA on the Golgi morphology, in noninfected cells in panels E, G, and H. (I to L) Localization of HRSV F and N proteins at 24 hpi without BFA treatment. (M to P) Dramatic changes in the localization of HRSV F (M) and N (O) proteins upon BFA treatment. (Q to T) The cell was treated with BFA for 5 hours, and then fresh medium was replaced without BFA; the sizes of the inclusion bodies (S) went back to being similar to the control (K), and it is possible to see filament formation budding from the plasma membrane, indicated by the arrows in panels Q, S, and T. The images represent a single focal plane in two independent experiments. Images were taken with Zeiss 780 confocal microscope. Magnification, ×63. All the scale bars = 10 μm.

To further explore the relationship between HRSV structural proteins and the Golgi complex, we treated infected cells with brefeldin A (BFA), a compound that binds Arf1-GDP protein and prevents attachment of coatomer protein complex I (COPI) to the Golgi. BFA treatment is known to reversibly induce Golgi stack disassembly and membrane redistribution to the ER (23, 24). The expected effect of BFA was observed in HEp-2 cells (Fig. 4A to H). Golgi disassembly induced by BFA in HRSV-infected cells led to reduced staining of both HRSV F and N proteins at the cell periphery (Fig. 4I to P). Also, the inclusion bodies appeared smaller in the presence of BFA (Fig. 4K and O and Fig. S2A to F), and the numbers of aggregates stained by HRSV N were significantly diminished in the presence of this drug (Fig. 4K and O and Fig. S2G to I and S3A to U). To reinforce that the phenotype observed was due to the disassembly of the Golgi, BFA was washed out after 5 hours of incubation, and the cells were incubated with fresh medium until 24 hpi. Under these conditions, the normal Golgi apparatus display was recovered, the inclusion bodies became larger, and labeling of the cell periphery filaments of the HRSV F and N proteins was restored (Fig. 4Q to T, arrows). Since our results showed that the BFA had impact in the inclusion body size, we wanted to know if this impact was modulated depending on the timing of BFA addition. To investigate this, HEp-2 cells were infected with HRSV, and at 4, 8, 12, and 20 hpi, the BFA was added and kept in the cell medium. All the cells were fixed in 4% paraformaldehyde (PFA) after 24 hpi and stained for HRSV N and giantin (Golgi), and the size of the inclusion bodies was measured (Fig. S3A to U). Interestingly, the size of inclusion bodies was dependent on the time of BFA addition (Fig. S3A to U), and they were significantly different from the control (Fig. S3U). The colocalization graph showed that the treatment with BFA caused a lower colocalization of HRSV N with giantin when BFA was kept for 20 hours (Fig. S3V). The colocalization of N with giantin increased when BFA was kept for 12 and 16 hours. This was likely due to the giantin dispersion and to the number of HRSV N aggregates. Of note, these experiments do not enable us to state that BFA acts directly on the coalescence of inclusion bodies or impairing the movement of the N protein that is associated with the Golgi. Taken together, these results suggest that normal Golgi integrity is required to deliver both glycosylated F and nonglycosylated N proteins to the cell periphery/surface and that the inclusion body size is affected by BFA treatment in unknown ways.

10.1128/mBio.01869-20.2 FIG S2 Effect of brefeldin A on the size and quantity of HRSV inclusion bodies. (A to C) IF for HRSV N protein in HRSV-infected cells not treated with brefeldin A. (D to F) IF for HRSV N protein in HRSV-infected cells treated with brefeldin A. (G to I) Quantity of HRSV aggregate/IB structures counted in HRSV-infected cells treated or not with brefeldin A, exemplified by panels H and I. All the images were taken at 24 hpi and are representative of a single focal plane from a Zeiss 780 confocal microscope. Magnification, ×63. The graph was based on the counting of at least 12 fields from two different experiments. The statistical method used was Student’s *t* test. *, *P* < 0.05; **, *P* < 0.01; ***, *P* < 0.001. All the scale bars = 10 μm. Download FIG S2, PDF file, 0.3 MB. Copyright © 2020 Cardoso et al. 2020 Cardoso et al. This content is distributed under the terms of the Creative Commons Attribution 4.0 International license.

10.1128/mBio.01869-20.3 FIG S3 The size of inclusion bodies is affected by the time of exposure to BFA. HEp-2 cells were infected with HRSV, and at 24 hpi they were fixed and stained for DAPI, HRSV N, and giantin. The IF images in the far-right column are merges of those in the three columns on the left. The figures are a representation of two independent experiments, and at least five fields of each experiment were used to measure the inclusion bodies. (U) Graph comparing the size of the inclusion bodies in HRSV-infected cells exposed or not to BFA during different time lengths. (V) Mander’s colocalization between HRSV N and giantin in HRSV-infected cells treated with BFA or DMSO (control). The panel U and V *x* axes depict the time lengths of exposure to BFA. The fluorescence images were taken with a Zeiss 780 confocal microscope. Magnification, ×63.The statistical method used in panels U and V was ANOVA one-way Tukey’s multiple-comparison test. *, *P* < 0.05; **, *P* < 0.01; ***, *P* < 0.001. All the scale bars = 10 μm. Download FIG S3, PDF file, 0.4 MB. Copyright © 2020 Cardoso et al. 2020 Cardoso et al. This content is distributed under the terms of the Creative Commons Attribution 4.0 International license.

### HRSV M and N proteins colocalize and interact with the early endosome protein sorting nexin 2.

**FIG 5:**
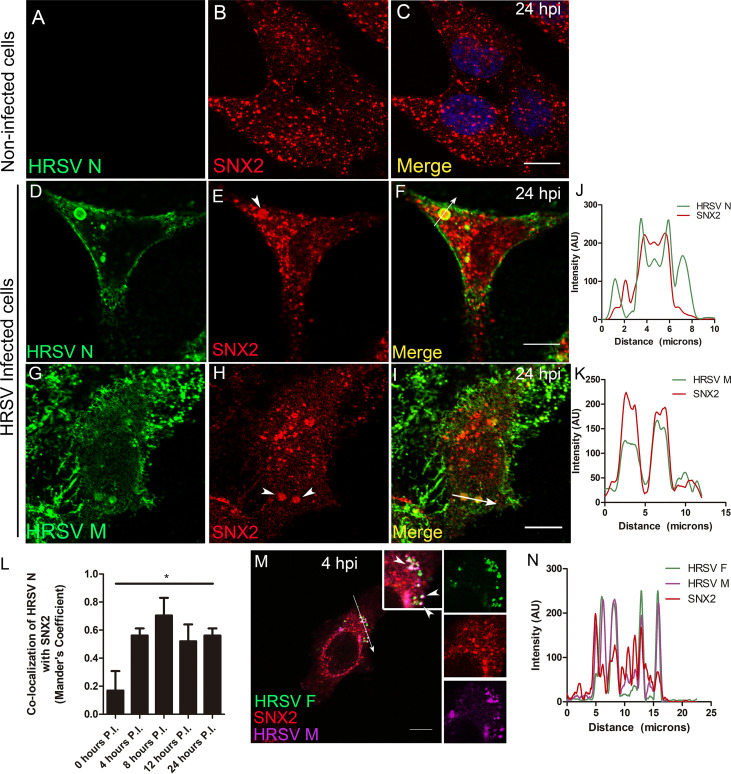
HRSV M and N proteins colocalize with the early endosome marker SNX2. (A to C) Staining for SNX2 in noninfected HEp-2 cells. (D and E) Separate channels for N protein and SNX2. (F) Colocalization of HRSV N protein and SNX2. (G and H) Separate channels of HRSV M and SNX2, with arrowheads indicating points of accumulation of SNX2. (I) Colocalization of HRSV M protein and SNX2. (J) Plot profile based on the arrow traced on panel F. (K) Plot profile traced on panel I. Images in panels A to I were taken at 24 hours postinfection. (L) Mander’s coefficient of colocalization of HRSV N with SNX2 over time postinfection; the asterisk indicates that the colocalization was significantly higher at 8 hpi than at all other time points (*t* test was used to compare times pairwise). (M) Triple colocalization of HRSV F and M proteins with SNX2 (arrowheads) at 4 hours postinfection. (N) Plot profile of the arrow traced on panel M. These figures are representative of at least three independent experiments and represent a single focal plane, taken with a Leica SP5 confocal microscope. The graph in panel L is representative of Mander’s colocalization of N with SNX2 from Z-stack imaging from at least three independent experiments. The *P* value was determined using ANOVA one-way Tukey’s multiple-comparison test. *, *P* < 0.05; **, *P* < 0.01; ***, *P* < 0.001. All the scale bars = 10 μm.

**FIG 6:**
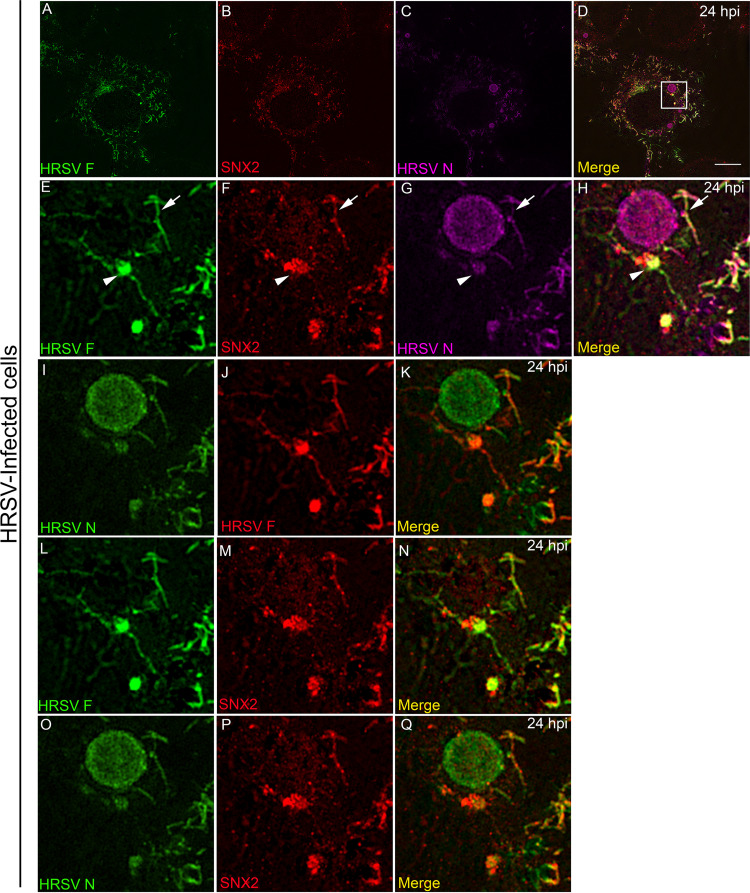
Superresolution image of SNX2 associated with HRSV inclusion bodies and intracellular filaments and vesicles containing HRSV proteins. (A to D) Superresolution view of an HRSV-infected cell. Panels A to C show separate channels for HRSV F, SNX2, and HRSV N. (E to H) Higher magnification of the inset in panel D in separate channels, with arrowheads pointing to HRSV F protein in vesicles and intercellular filaments. It is possible to see filaments containing SNX2 and HRSV F protein (H) and (N) and filaments containing SNX2 and HRSV N protein (H) and (K), indicated by arrows. SNX2 is associated with inclusion bodies in panels F, H, N, P, and Q. All images were taken at 24 hpi and are representative of a single plane from Z-stack imaging, taken with Nikon N-SIM microscope (superresolution imaging). All the scale bars = 10μm.

**FIG 7:**
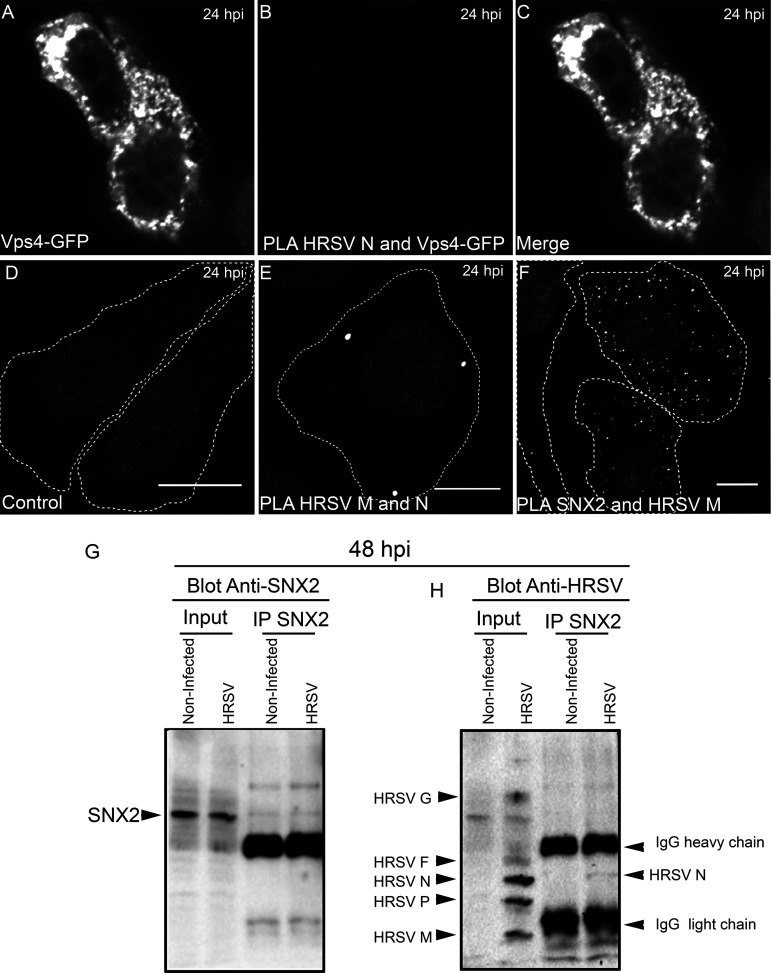
HRSV M and N proteins interact with SNX2. (A) HEp-2 cells transfected with Vps4-GFP and infected with HRSV. (B) PLA for HRSV N and GFP in the cells depicted in panel A. (C) Merge of panels A and B. (D) PLA control where HRSV-infected cells were stained only with the HRSV M primary antibody, omitting the anti-SNX2 primary antibody. (E) Interaction between HRSV M and N proteins in infected cells with white dots corresponding to PLA signal. (F) Interaction between HRSV M and SNX2 proteins, with white dots corresponding to PLA signal. (G and H) Coimmunoprecipitation of SNX2 in HEp-2 cells at 48 hpi. In panel G, the membrane was blotted with rabbit anti-SNX2 antibody, and in panel H, with goat anti-HRSV polyclonal antibody, showing that SNX2 coimmunoprecipitated with HRSV N protein (arrowhead). All PLA images were taken at 24 hpi and are representative of a single focal plane from a Zeiss 780 confocal microscope. Magnification, ×63. The percentage for HRSV M and SNX2 PLA positive cells was approximately 45%. This counting was obtained from at least six fields in two different experiments. All PLA results are representative of two independent experiments. All the scale bars = 10 μm.

To assess whether HRSV N and M proteins are targeted to early endosomes, immunofluorescence (IF) microscopy for sorting nexin 2 (SNX2) was performed in HEp-2 cells at different times pi. SNX2 plays an important role in the formation of the retromer coat by shaping tubular structures out of the membranes of early endosomes (25), and therefore, it is an appropriate early endosome marker. Different from the normal distribution of SNX2 in noninfected cells (Fig. 5A to C), there was evident colocalization of SNX2 with HRSV N (Fig. 5D to F and J) and M (Fig. 5G to I and K) at 24 hpi. Notably, SNX2 appeared accumulated in the inclusion bodies as confirmed by the plot profiles (Fig. 5J and K). Furthermore, the Mander’s coefficient of colocalization of HRSV N protein with SNX2 was determined at different times pi (Fig. 5L), and the peak of colocalization happened at 8 hpi. To investigate if the nonglycosylated proteins reach endosomes at early stages of infection when inclusion bodies are not apparent, IF was performed for HRSV F, M, and SNX2 at 4 hpi. The experiment revealed that while HRSV F and SNX2 have a better colocalization, HRSV M protein partially colocalized in some SNX2-containing vesicles (Fig. 5M and N), suggesting that part of the HRSV nonglycosylated M proteins are localized in early endosomes at that time of the replication cycle. Seeking a better understanding of the relationship between SNX2 and HRSV inclusion bodies, superresolution imaging was performed for HRSV N and SNX2 (Fig. 6A to Q). It was apparent that SNX2 was mostly located at the edges, but to a lesser extent also within HRSV inclusion bodies (Fig. 6D, H, and Q). In agreement with the data in Fig. 5, once again, HRSV F and N were seen in the same SNX2-containing vesicles (Fig. 6E to H, arrowheads). It is noteworthy that intracellular filaments appeared to be emerging from the same small protein aggregates that contain HRSV F and N proteins and SNX2 (Fig. 6E to H, arrows). As superresolution imaging demonstrated tight proximity between HRSV N and SNX2, PLA was performed to check for a possible interaction between SNX2 and HRSV M protein (Fig. 7). Because HRSV M and N proteins are known to interact with one another (26), they were used as positive controls for the PLA, revealing points of fluorescence distributed throughout the cytoplasm (Fig. 7E). Interestingly, PLA for HRSV M and SNX2 showed abundant intracellular puncta dispersed throughout the cytoplasm (Fig. 7F). To confirm that HRSV proteins interact with SNX2 by a different approach, a coimmunoprecipitation assay was performed using an anti-SNX2 antibody. A band corresponding to HRSV N protein was coimmunoprecipitated with SNX2 (Fig. 7D and E, arrowhead). Taken together, the results suggest that HRSV structural proteins interact with SNX2 and may recruit this host protein to virus-induced compartments during assembly.

### HRSV selectively recruits endosomal proteins to viral inclusion bodies.

**FIG 8:**
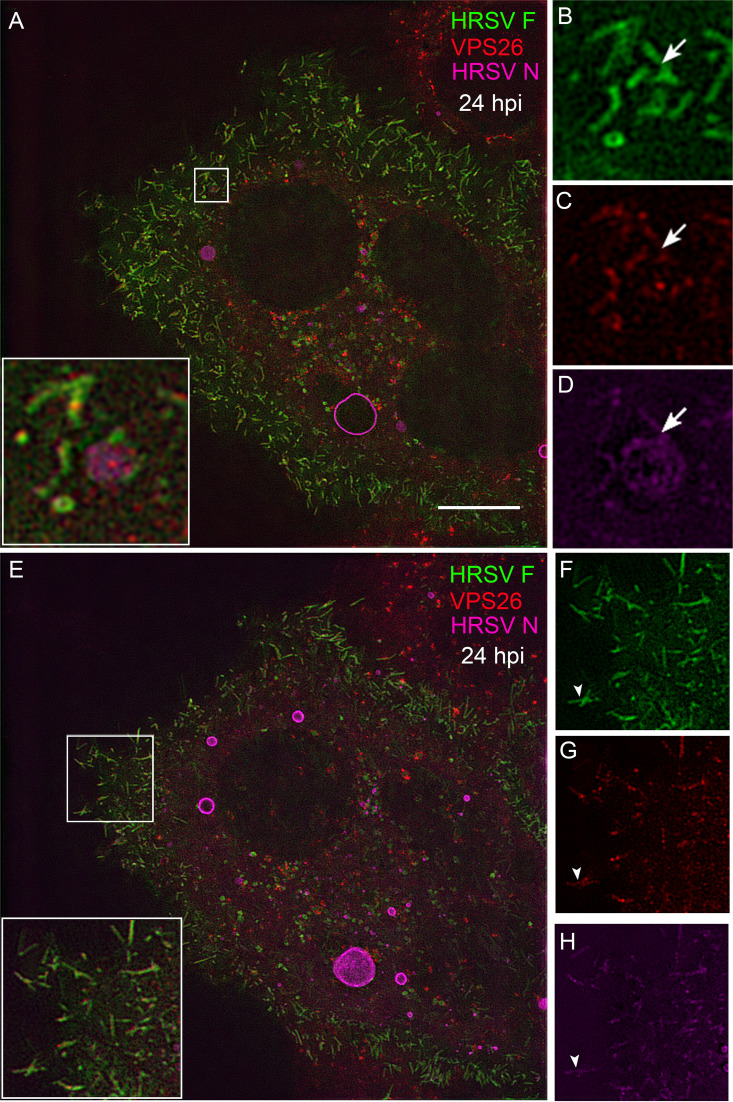
Superresolution imaging of the Vps26 component of the retromer colocalizing with HRSV F and N. (A) Overview of HRSV-infected HEp-2. (B to D) Separate channels of the inset in panel A with arrows pointing to intracellular filament-shaped structures emerging from the same vesicle containing HRSV F (B), Vps26 (C), and HRSV N (D). (E) Overview of the same cell in panel A in a different Z plan. (F to H) Separate channels of the inset in panel E with arrowheads pointing to Vps26 in filament-shaped structures containing HRSV proteins in the plasma membrane. All images were taken at 24 hpi and are representative of a single plane from Z-stack imaging. The images were taken with a Nikon N-SIM microscope (superresolution imaging). All the scale bars = 10 μm.

The association of SNX2 with SNX1, Vps26, Vps29, and Vps35 forms the retromer complex, a vesicle coat involved in retrograde transport of proteins from endosomes to the TGN (27). In order to verify whether another retromer component, besides SNX2, is present in structures containing HRSV proteins, IF for Vps26 was performed in HRSV-infected cells at 24 hpi (Fig. 8). Although less intense than for SNX2, and not localized in larger inclusion bodies (Fig. 5 and 6), colocalization of N with Vps26 was clearly visible (Fig. 8A to D). Interestingly, Vps26 colocalization was restricted to tubular structures containing HRSV F and N proteins. These structures could be either tubular endosomal structures (Fig. 8A to D, arrows) or filaments protruding from the plasma membrane (Fig. 8E to H, arrowheads). Importantly, the results indicate that more than one component of the retromer is enriched in structures containing HRSV proteins. Colocalization between HRSV and retromer proteins may be explained by either the recruitment of these host proteins from the cytosol to virus-induced compartments or by the targeting of HRSV proteins to early endosomes. Therefore, to verify whether early endosome elements other than Vps26 were recruited to inclusion bodies, HRSV-infected cells were stained for the early endosome antigen-1 (EEA1). Different from what was observed for SNX2, EEA1 did not colocalize with HRSV inclusion bodies in infected cells at 24 hpi (Fig. S4A to I). This indicates that there is specific relocalization of SNX2 to HRSV inclusion bodies, independent from other early endosome elements.

10.1128/mBio.01869-20.4 FIG S4 The early endosome marker EEA1 does not colocalize with HRSV inclusion bodies. (A to C) Noninfected HEp-2 cells with labeling for EEA1 (B) and SNX2 (C). (D) Merge of panels A, B, and C. (E to H) HRSV-infected cells at 24 hpi, labeled for HRSV N protein (E), EEA1 (F), and SNX2 (G). The arrowheads show that EEA1 marker (E) does not colocalize with HRSV N in the inclusion bodies shown in panels E, F, and H, differently from SNX2 (G), which colocalizes with HRSV N (E, G, and H). (I) Mander’s coefficient of colocalization between HRSV N with SNX2 and EEA1. All images were taken at 24 hpi and are representative of a single plane from a Z-stack. Magnification, ×63 with a Zeiss 780 confocal microscope. The statistics were acquired with Student’s *t* test. *, *P* < 0.05; **, *P* < 0.01; ***, *P* < 0.001. All the scale bars = 10 μm. Download FIG S4, PDF file, 0.3 MB. Copyright © 2020 Cardoso et al. 2020 Cardoso et al. This content is distributed under the terms of the Creative Commons Attribution 4.0 International license.

### Fractionation of HRSV-infected cells reveals repositioning of SNX2 in HRSV-infected cells.

**FIG 9:**
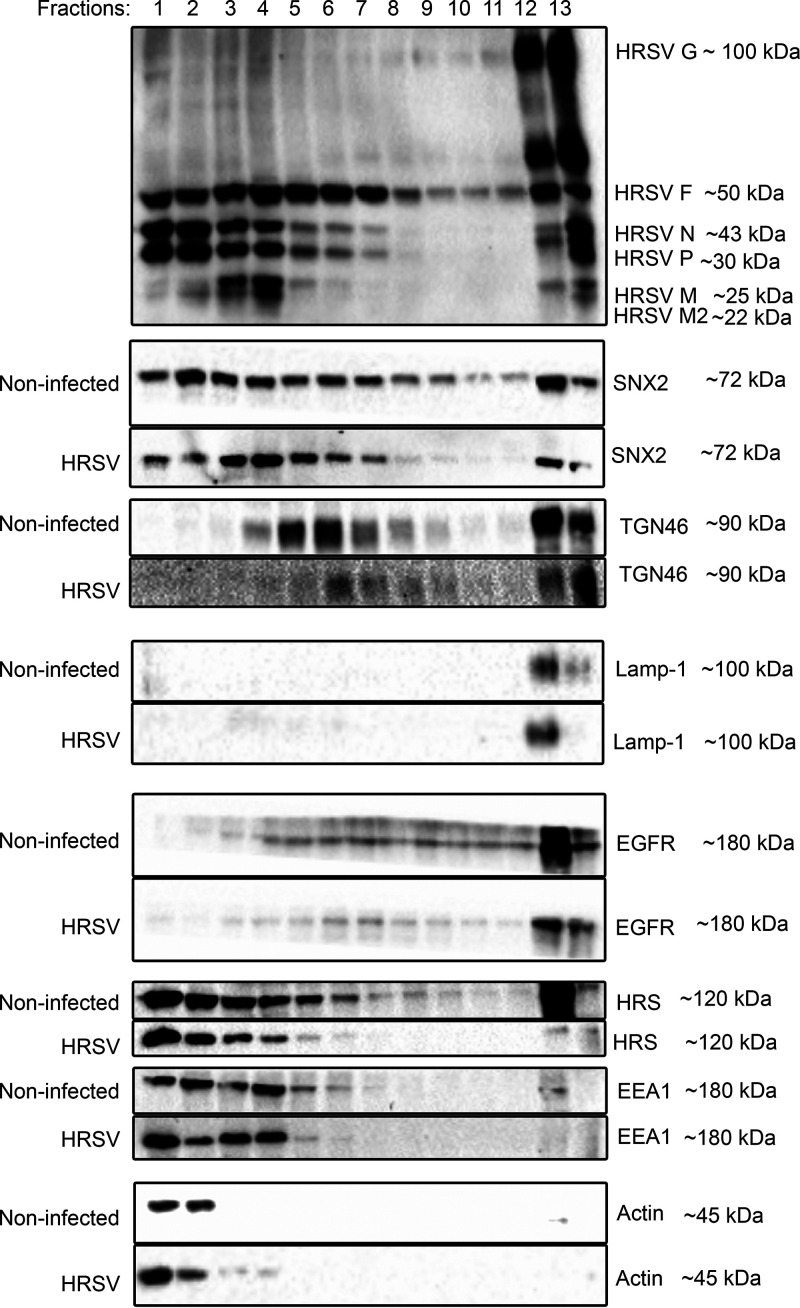
Cell fractionation. HEp-2 cells uninfected or infected (HRSV) were harvested at 24 hpi, lysed, and loaded on the top of a discontinuous 12 to 30% glycerol gradient. After ultracentrifugation, fractions were collected and analyzed by SDS-PAGE and Western blotting. In HRSV-infected cells, SNX2 accumulated in the fractions of HRSV nonglycosylated proteins (fractions 1 to 7), whereas in noninfected cells, SNX2 was detected in all fractions. In HRSV-infected cells, the TGN46 protein is more abundant in the last fraction corresponding to the plasma membrane. Lamp-1 was not detected in the same fractions that accumulated HRSV N, P, M, and M2 proteins. Detection of EEA1 was similar between HRSV-infected and noninfected cells. Actin and EGFR are controls of cytosol and plasma membrane, respectively. The figure is a representation of two experiments. The membranes were visualized using a Bio-Rad Chemidoc.

To verify the partitioning of SNX2 in different compartments of HRSV-infected cells, cellular fractionation and Western blotting were performed. In HRSV-infected cells, SNX2 accumulates in fractions 1 to 7, which correspond to those that contain the nonglycosylated HRSV proteins that are known to compose the inclusion bodies (Fig. 9). This was in contrast to noninfected cells, in which SNX2 was detected throughout all the fractions. Interestingly, in HRSV-infected cells, TGN46 was detected in fractions 7 to 10, revealing a shift from the normal distribution seen in noninfected cells, in which TGN46 appeared in fractions 4 to 8, suggesting recruitment of TGN46 toward more dense fractions containing plasma membranes (Fig. 9). Importantly, the distribution of lysosomal-associated membrane protein-1 (Lamp-1), the early endosomal marker EEA1, and the plasma membrane protein EGFR appeared to be little affected by HRSV infection.

### TGN46 and SNX2 colocalize with HRSV M and N proteins in virus filaments on the cell surface.

**FIG 10:**
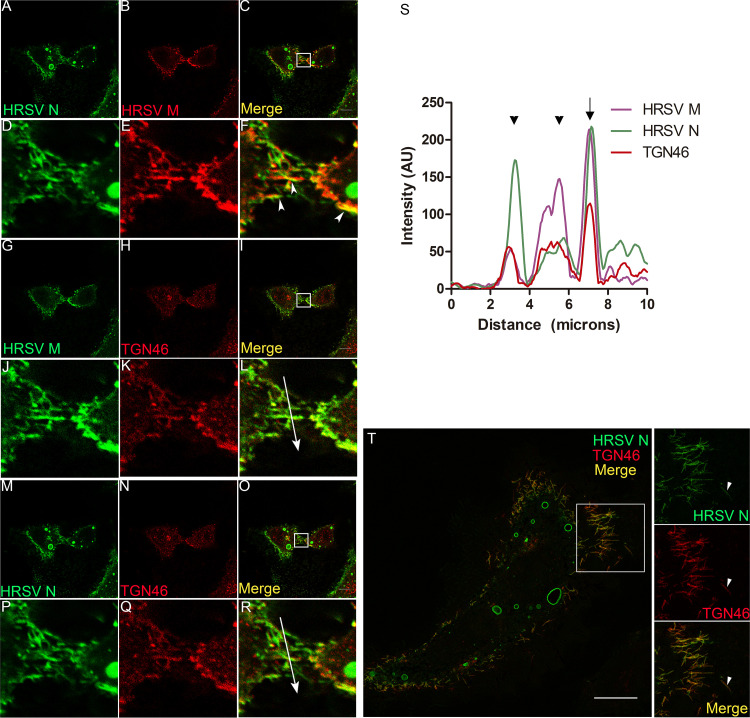
The trans-Golgi marker TNG46 is detected in HRSV filaments in HEp-2 cells. (A and B) Separate channels of HRSV N and M. (C) Colocalization of HRSV N and M proteins. (D, E, and F) Higher magnifications of panels A, B, and C, respectively, corresponding to the area indicated in panel C, with arrowheads pointing to HRSV budding filaments on the cell surface. (G and H) Separate channels of HRSV M and TGN46. (I) Colocalization of HRSV M and TGN46 in the cells shown in panel C. (J, K, and L) Higher magnifications of panels G, H, and I, respectively, corresponding to the area indicated in panel I. (M and N) Separate channels of HRSV N and TGN46. (O) Colocalization of HRSV N and TGN46. (P, Q, and R) Higher magnifications of panels M, N, and O, respectively, corresponding to the area indicated in panel O. (S) Plot profile of the colocalizations of HRSV M and N proteins with TGN46, the arrows traced in panels L and R; the arrow points to a perfect correlation in the plot profile. (T) Superresolution image of an HRSV-infected cell, with arrowheads pointing to filaments budding from the cell, containing HRSV N and TGN46. All the images were taken at 24 hpi. Panels A to S are representative of a single plane from Z-stack imaging or a single focal plane of at least three independent experiments taken with a Leica SP5 confocal microscope. Magnification, ×63. Panel T was taken with a Nikon N-SIM microscope (superresolution imaging) and represents a single focal plane from Z-stack imaging. All the scale bars = 10 μm.

**FIG 11:**
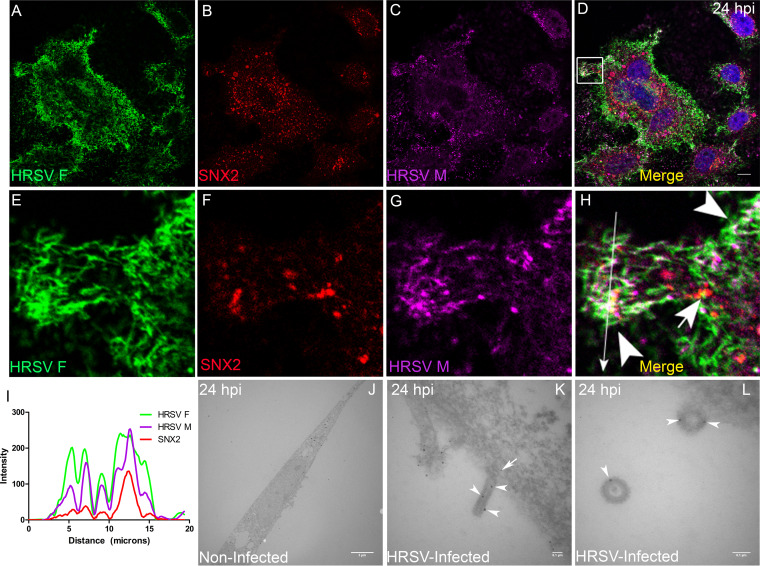
The retromer SNX2 protein is detected in HRSV filaments. (A to C) Separate channels for HRSV F, SNX2, and HRSV M. (D) Colocalization of HRSV F and M proteins with SNX2. (E to H) Higher magnifications of panels A, B, C, and D in the area delimited in panel D. Panel H shows triple colocalization of HRSV F, SNX2, and HRSV M proteins, with arrowheads pointing to HRSV filaments and the arrow pointing to an SNX2 vesicle partially colocalizing with HRSV F protein. (I) Plot profile of the colocalizations of HRSV F, M, and SNX2 corresponding to the long arrow traced on panel H. (J) Electron microscopy of a noninfected HEp-2 cell. (K) Detail of a virus filament with SNX2 detected by immunogold labeling along the filament (arrowheads) and in the basis (arrow). (L) Higher magnification showing HRSV-like structures with the presence of SNX2 (arrowheads). The figures are representative of a single focal plan taken with a Leica SP5 microscope. Magnification, ×63. The experiment was repeated at least three times. Scale bars of immunofluorescence images = 10 μm. The electron microscopy figures were taken in a Jeol JEM-100 CXII transmission electron microscope and are representative of one experiment. The size of the scale bars of the electron microscopies are shown in the images.

In keeping with previous observations that HRSV budding forms typical filamentous shapes on the plasma membrane (28, 29) that contain HRSV M and N proteins (Fig. 10A to F, arrowheads), TGN46 colocalized extensively with those viral proteins in the context of such filaments (Fig. 10G to S). This was reinforced by superresolution microscopy of filament-shaped structures on HRSV-infected cells (Fig. 10T), showing an abundant localization of the TGN46 in the filamentous structures containing the HRSV proteins. Curiously, some of those filaments appeared to be detaching from the cells (Fig. 10T, arrowheads). SNX2 also colocalized with HRSV structural proteins F and M in viral filaments on the cell surface (Fig. 11A to I). Immunogold electron microscopy confirmed the presence of SNX2 in filament-shaped structures at the plasma membrane, as well as in structures apparently budded from HRSV-infected cells (Fig. 11J to M). Together, these results suggest that the HRSV particles may contain SNX2 and TGN46 to some extent.

### Silencing of sorting nexins 1 and 2 partially impairs HRSV production and syncytium formation.

**FIG 12:**
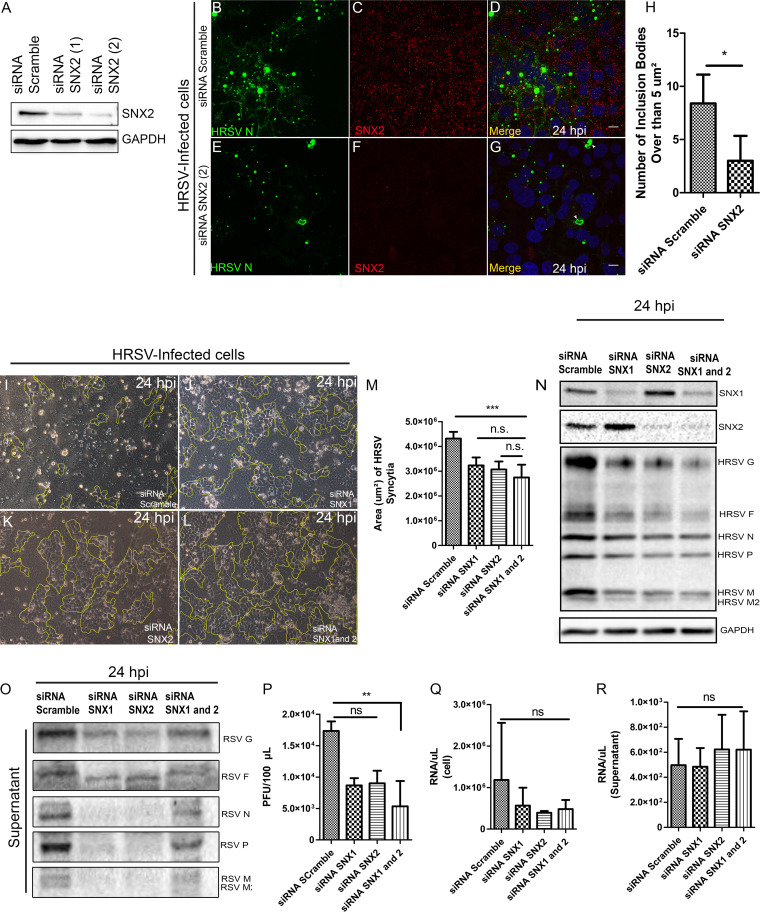
Small interfering RNA for SNX1 and 2. (A) Immunoblotting for SNX2 in cells treated with siRNA for SNX2 after two shots (1 and 2) done with the same amount of siRNA; GAPDH was the load control. (B to D) Panel D shows a merge of panels B and C in cells treated with siRNA scrambled. (E to G: Panel G shows a merge of panels E and F in cells silenced for SNX2; arrowheads point to irregular-shaped inclusion bodies. (H) Graph of the inclusion body quantity over than 5 μm^2^ found in cell siRNA scramble and siRNA for SNX2 24 hpi. (I) Monolayer of HEp-2 cells, infected by HRSV and treated with scrambled siRNA. (J, K, and L) HRSV-infected HEp-2 monolayers treated with siRNA for SNX1, SNX2, and SNX1 and 2, respectively. The markings in the figures outline cell islands without syncytium formation. (M) Graph showing the sizes of HRSV-induced syncytia in cells treated with scrambled siRNA and siRNAs for SNX1, SNX2, and SNX1 and 2 at 24 hpi. (N) Immunoblotting for SNX1, SNX2, or both and for HRSV proteins, using HRSV-infected HEp-2 cells treated with scrambled siRNA or siRNA for SNX1, SNX2, or SNX1 and 2 at 24 hours postinfection. (O) Immunoblotting for HRSV proteins, using supernatants of HRSV-infected HEp-2 cells treated with scrambled siRNA or siRNA for SNX1, SNX2, or SNX1 and 2, at 24 hours postinfection. (P) HRSV progeny production in infected cells treated with scrambled siRNA or siRNA for SNX1, SNX2, or SNX1 and 2. (Q and R) HRSV RNA genome copies at 24 hours postinfection in cells and supernatants, respectively, in cultures treated with scrambled siRNA or with siRNA for SNX1, SNX2, or SNX1 and 2. The images are representative of a single focal plan, taken with a Zeiss 780 microscope. Magnification, ×63. This set of figures is representative of three independent experiments. The statistic of panel H was done using Student’s *t* test, and the graphs in panels M, P, Q, and R were done using ANOVA one way Tukey’s multiple-comparison test. *, *P* < 0.05; **, *P* < 0.01; ***, *P* < 0.001; ns, nonsignificant.

Once it was demonstrated that SNX2 colocalizes and interacts with HRSV proteins, a knockdown (KD) experiment was done using small interfering RNA (siRNA) for sorting nexins 1 and 2 in HEp-2 cells (Fig. 12). It is well known that SNX1 and 2 form heterodimers; therefore, siRNA was performed for either SNX1 or SNX2 alone, and for both at the same time, to check for the impact of their silencing in the HRSV replicative cycle. The IF for HRSV N protein at 24 hpi demonstrated that the quantity of the inclusion bodies larger than 5 μm^2^ were slightly lower in cells KD for SNX2, than in those treated with the scrambled siRNA control (Fig. 12A to H). We chose 5 μm^2^ as the threshold because Rincheval et al. (18) recently demonstrated that the HRSV mature inclusion bodies were larger than 5 μm^2^. In addition, cells KD for SNX2 displayed a notable reduction of staining for HRSV N protein in the cell periphery (Fig. 12D and G, arrowheads). Moreover, although syncytium formation in cells silenced for SNX1, SNX2, or SNX1 and 2 was not completely abrogated, there were significantly fewer syncytia in cells silenced for each or both SNX proteins (Fig. 12J to M), than in cells treated with scrambled siRNA (Fig. 12I and M). In agreement with the reduction in syncytium formation, Western blot analysis of HEp-2 cells at 24 hpi showed that the intracellular levels of HRSV G, F, and M proteins were reduced in cells KD for SNX1, SNX2, or SNX1 and 2 in comparison with the control (Fig. 12N). In addition, the immunoblotting of siRNA-treated cells demonstrated a reduction in HRSV protein levels in the supernatants (Fig. 12O). Consistently, there was a significant reduction in HRSV progeny production in cells silenced for SNX1 and 2 at 24 hpi (an approximate 70% reduction in the double KD) (Fig. 12P). Nevertheless, the viral genomic RNA production quantified at 24 hpi was not significantly altered in cells and supernatants upon KD of SNX1, SNX2, or SNX1 and 2 (Fig. 12Q and R), indicating that viral RNA replication was not significantly affected by the reduction in SNX1 and 2 levels in infected cells. Taken together, these data led us to conclude that the KD for the SNXs in HRSV-infected cells had an impact on the quantity of mature inclusion bodies, syncytium formation, HRSV protein amounts, and progeny production.

## DISCUSSION

It is well known that the HRSV envelope glycoproteins G, F, and SH traffic through the Golgi apparatus, where the F protein undergoes maturation cleavages (2). However, little is known about the traffic of the HRSV nonglycosylated proteins to virus assembly sites. The present study has shown that the nonglycosylated HRSV structural proteins M and N are detected in association with the secretory pathway and retromer elements, and these are novel findings that contribute to understanding the HRSV assembly process.

In the present study, HRSV M and N proteins partially colocalized with giantin, a specific marker for cis and medial Golgi, mainly at the edges of this organelle. This finding that the traffic of the M protein is associated with membranes is in agreement with results by Henderson et al. (30), who reported an association of the M protein with membrane-enriched fractions of HRSV-infected cells. We have also shown that the HRSV F protein, in the Golgi, partially colocalized with inclusion bodies located at the vicinity of cis and medial Golgi cisternae. Since the N terminus of HRSV F glycoprotein is in the lumen of the Golgi (31), the cytosolic C-terminal portion of F could interact with the nonglycosylated HRSV M and N proteins within inclusion bodies. This was reinforced by results of superresolution microscopy and PLA (Fig. 2) and agrees with what was previously proposed by Ghildyal et al. (32), who suggested that the HRSV G and M proteins interact with one another in the Golgi. Céspedes et al. (14) also addressed the issue of HRSV N protein colocalization with the cis and medial Golgi using another Golgi marker, GALNT2. In the present study, we have expanded that by showing that in addition to the cis and medial Golgi, HRSV M and N proteins only partially colocalized with the TGN46, and this colocalization was significantly less than that of HRSV F with TGN46.

The importance of the Golgi apparatus in the HRSV protein traffic was also supported by experiments in which cell treatment with BFA resulted in smaller HRSV inclusion bodies and reduced N and F proteins in the cell surface, an effect that was reversible by BFA wash out. These observations are also in keeping with data by Céspedes et al. (14), who showed a BFA-induced reduction of N protein in the plasma membrane by flow cytometry. The present study added to that by showing that both HRSV glycosylated F and nonglycosylated N proteins depend on the integrity of the Golgi to traffic to the plasma membrane. Even though we are prone to believed that the lower quantity and size of inclusion bodies in BFA-treated cells is due to the dependency of the Golgi for its traffic, it is not possible to conclude that the inclusion body size was impacted only because of the Golgi disassembly. These phenomena could be due to the effect of BFA directly on the inclusion bodies, and an in-depth study should be done to fully understand it. Another explanation for the partial colocalization of HRSV inclusion bodies with giantin is that, in the HRSV entry process, vesicles containing the virus reach intracellular places near the Golgi region, and perhaps this is the first place where the inclusion bodies will appear, and therefore, as they get bigger they happen to colocalize with the Golgi. However, once again, this does not explain why the inclusion body size and delivery to the plasma membrane are altered by BFA treatment.

It has been previously shown that the HRSV N protein reaches late endosomes, as indicated by colocalization of N with LAMP1, a late endosome marker (14). The present study showed a considerable colocalization of HRSV M and N proteins with SNX2, which is a protein commonly found in association with early endosomes (Fig. 5). This suggests that HRSV inclusion bodies that concentrate nonglycosylated N and M proteins are sites of preferential accumulation of SNX2. Also, the accumulation of SNX2 in HRSV inclusion bodies is not uniform, as seen in Fig. 5E and H, and Fig. S4. We do not know yet what that means; however, Rincheval et al. (18) recently demonstrated that inclusion bodies are dynamic and heterogeneous, and this different pattern of SNX2 accumulation could be due to the inclusion bodies’ heterogeneity and maturation during the course of the HRSV infection.

The HRSV N and M proteins are known to interact with one another (2, 26), which was confirmed by the present PLA results. Also, the present study has shown that the HRSV M and N proteins interact with SNX2, as evidenced by PLA for the M protein, and immunoprecipitation for the N protein. The findings of the present study indicate that there is an association of HRSV nonglycosylated proteins with elements of the secretory pathway. This observation is in agreement with previous demonstration of the recruitment of the adaptor protein complex-3 (AP-3), known to be involved in protein traffic between endosomes and the Golgi (33–35), to HRSV inclusion bodies and its interaction with the HRSV M protein (36). Furthermore, SNX2 was shown to colocalize with the AP-3 complex, which in turn, colocalizes with Vps26 (34, 37). Moreover, a recent study has pointed out the colocalization of HRSV glycosylated G and nonglycosylated proteins in the same vesicles as the glycoprotein G recycles back from the plasma membrane (38).

It was noteworthy that the PLA of the HRSV M protein with SNX2 was seen as numerous puncta throughout the cytoplasm of infected cells. While our results do not confirm that all the PLA dots of M and SNX2 colocalized with HRSV inclusion bodies, considering that they were much more abundant than the viral inclusion bodies at the same time postinfection, it is tempting to speculate that the HRSV M protein traffics through early endosomes before reaching the larger more mature inclusion bodies or the plasma membrane. Since the HRSV M protein is a link between glycosylated and nonglycosylated proteins to form viral particles, its trafficking in association with endosomes could be a help to bridge the glycosylated proteins with RNP-complexed N protein in an endosome, which would facilitate the viral traffic and assembly. However, more studies should be performed to test this hypothesis, such as, for example, expression of the F, M, and N proteins in different combinations to check their capacity to associate with membranes *in vitro*. In addition, this movement of M protein is reminiscent of what was proposed by Cifuentes-Muñoz et al. (39), who showed that the formation of inclusion bodies of human metapneumovirus, another virus in the family *Pneumoviridae*, is dynamic, resulting from the coalescence of smaller elements.

It has been previously shown that in HRSV-infected cells, long filamentous structures containing HRSV proteins protrude from intracellular vesicles toward the cytosol (38). Our results showed that such intracellular filaments contain SNX2, suggesting that SNX2 may be involved in their formation. SNX2 and Vps26 are known components of the retromer complex that shapes filaments out of membranes (37), and we have detected Vps26 and SNX2 in vesicles containing HRSV N and F proteins as well as in filamentous structures pinched out from their surfaces, which reinforces that retromer components can be present in vesicles and filaments containing HRSV proteins.

It is well known that HRSV traffic is endosome dependent (2), but exactly how the HRSV nonglycosylated proteins interact with endosomes is not understood. Considering that SNX2 and Vps26 were detected in vesicles containing both glycosylated and nonglycosylated HRSV proteins, we asked if these vesicles could be early endosomes. We did not find detectable recruitment of EEA1, a marker of early endosomes, to HRSV inclusion bodies (Fig. S3), suggesting that SNX2 recruitment to inclusion bodies is selective and that inclusion bodies are not canonical early endosomes. Corroborating these data, cell fractionation showed recruitment of SNX2 to fractions that correspond to inclusion bodies, while EEA1 distribution in cell fractions was not different between HRSV infected and noninfected cells. Moreover, Lamp1, a marker of late endosomes and lysosomes, was very little detected in fractions corresponding to HRSV inclusion bodies, suggesting that these organelles are not components of HRSV inclusion bodies. It is noteworthy that actin was concentrated in cell fractions corresponding to HRSV inclusion bodies, in agreement with previous findings by Shahriari et al. (40).

The results of immunofluorescence and cell fractionation experiments indicate that TGN46 accumulated in the last fraction, which is enriched for plasma membrane (Fig. 9 and 10) in HRSV-infected cells compared to noninfected cells. In addition, TGN46 was shown to be present in HRSV at the plasma membrane by superresolution microscopy, including seemingly detached HRSV buds. Taken together, these results suggest that TGN46 is carried in budded virus particles. Fluorescence findings similar to those obtained for TGN46 were observed for SNX2, which was detected in filamentous structures budding from the plasma membrane. It is interesting also that not all filaments containing HRSV proteins contained TNG46 and SNX2, suggesting that the particles that bud from the plasma membrane are not homogeneous.

One should keep in mind that TGN46 recycling from the plasma membrane back to the trans-Golgi could be compromised in HRSV-infected cells, since SNX2, which is part of the retromer complex, is recruited by inclusion bodies.

Importantly, the knockdown of SNX1 and 2 had a negative impact on several HRSV processes. First, upon cell silencing for these two proteins, alone or in combination, the amounts of intracellular HRSV F, G, and M proteins were reduced, and the HRSV inclusion bodies were smaller than in control cells at 24 hpi. Moreover, the knockdown for SNX1, 2, or both resulted in significantly smaller syncytia (Fig. 12). While the knockdown for sorting nexins did not affect HRSV replication as indicated by genome copy quantities at 24 hpi, the titers of infectious virus in the supernatant were significantly reduced in cells simultaneously silenced for SNX1 and 2. It is possible that the silencing of SNX1 and 2 may have altered the final content of structural proteins in progeny virions compared to controls, which might have affected the quantification of progeny production by plaque assay. These findings should be interpreted with caution since previous studies have shown that even HRSV particles lacking envelope G and SH glycoproteins retain infectivity (12, 13), which could help to explain the moderate reduction seen in virus titers produced in cells silenced for SNX1, SNX2, and SNX1 and 2. SNX1 and 2 are part of the retromer complex, which is involved in the transport of cargo from endosomes to the trans-Golgi network (41). It has also been shown that in the absence of SNX1 and 2, some proteins that depend on the retromer complex for their traffic are addressed to lysosomal compartments for degradation (25). This could explain why in cells silenced for SNX1 and SNX2, there was a reduction in amounts of F protein and smaller syncytia at 24 hpi.

In summary, the present study contributed findings that help to establish the importance of the secretory pathway in the traffic of HRSV nonglycosylated structural proteins and showed for the first time the involvement of retromer proteins in the biogenesis of HRSV particles.

## MATERIALS AND METHODS

### Cells and virus.

HEp-2 cells were grown in minimal essential medium (MEM) with 10% fetal bovine serum (FBS) and maintained in MEM with 2% FBS in 5% CO_2_ at 37°C. HRSV-A of the strain Long (ATCC VR-26) was propagated in HEp-2 cells, and the stock was titrated by plaque assay using routine methods (42).

### HRSV *in vitro* infection.

Infection assays were done on glass coverslips inside 24-well plates, using HRSV stock diluted in phosphate-buffered saline (PBS) to reach a multiplicity of infection (MOI) of 1, with 1 hour of incubation at 4°C to synchronize virus entrance. Then, coverslips were washed three times in cold PBS, replenished with MEM with 2% FBS at 37°C, and then incubated in 5% CO_2_ at 37°C. At 0, 4, 8, 12, 24, and 48 hpi the coverslips were fixed. For the immunoprecipitation and cell fractionation assays, HEp-2 cell monolayers were prepared in 75-cm^2^ flasks, infected at MOIs of 0.1 and 1, and harvested at appropriate times.

### Immunofluorescence.

At appropriate times, coverslips were fixed with 4% paraformaldehyde (PFA) in PBS for 10 minutes at room temperature and then washed once with PBS, permeabilized with 0.01% Triton in PBS for 20 minutes, and then blocked with PBS containing 3% bovine serum albumin (BSA) for 30 minutes. After 10 washes with PBS, coverslips were incubated with the primary antibody for one hour at 37°C and then washed 10 times with PBS and incubated with the secondary antibody for 1 hour at 37°C. Coverslips were washed 10 times in PBS and once in distilled water and then mounted on glass slides using Flouromount. Nuclei were stained with DAPI, and images were acquired on Leica SP5 or Zeiss 780 confocal microscopes or a Nikon N-SIM microscope (super resolution imaging) and were analyzed using ImageJ software Fiji.

### Antibodies.

The antibodies used for immunofluorescence (IF) were rabbit polyclonal anti-TGN46 (ABT95; Millipore), sheep polyclonal anti-TGN46 (Abd Serotec AHP500; Bio-Rad), rabbit polyclonal anti-SNX2 and anti-VPS26 (gifts from C. Haft, NIDDK, NIH) (43), Alexa Fluor 488-labeled mouse monoclonal antibody (MAb) anti-HRSV F protein (133-1H MAB8262X; Millipore), mouse MAb anti-HRSV N protein labeled with FITC (MAB858-3F N; Millipore), mouse polyclonal anti-HRSV M protein (44), rabbit polyclonal anti-giantin (PRB-114C-200; Covance), mouse MAb anti-RSV F protein (MAB8599; Millipore), rabbit polyclonal anti-Lamp-1 D2D11 XPR (9091T; Cell Signaling), mouse MAb anti-EEA1 (610457; BD), rabbit polyclonal HRS (ab155539), Alexa Fluor 594-labeled goat anti-rabbit (ab150080; Abcam), Alexa Fluor 647-labeled goat anti-mouse (ab150115; Abcam), Alexa Fluor 488-labeled donkey anti-mouse (A21202; Invitrogen), and Alexa Fluor 647-labeled donkey anti-sheep (A21448 Thermo Fisher).

### Transmission electron microscopy.

HEp-2 cells grown to 80 to 100% confluence were infected with HRSV at an MOI of 1, and at the appropriate time, the culture was fixed in a mixture containing 0.05% glutaraldehyde, 2% paraformaldehyde, and 0.025% calcium chlorate in 0.1 M cacodylate buffer pH 7.4 in a microwave oven for 7 seconds at maximum potency. Cells were then washed in 50 mM glycine in PBS for 15 minutes, incubated with 1% BSA in PBS for 45 minutes under gentle shaking, and then permeabilized with 0.05% saponin diluted in PBS with 1% BSA (PBS-BSA) for 30 minutes. Cells were then incubated with rabbit polyclonal anti-SNX2 antibody diluted in PBS with 1% BSA for 2 hours at room temperature under gentle shaking. After that, cells were washed four times for 5 minutes each with PBS containing 1% BSA, followed by incubation with anti-rabbit antibody conjugated with 6 nm colloidal gold (Jackson ImmunoResearch Laboratories, Inc.) for 1 hour at room temperature. Cells were then fixed with 2.5% glutaraldehyde in 0.1 M cacodylate buffer for 1 hour at room temperature, washed twice with cacodylate buffer, and soaked overnight in the same buffer. The next day, cells were washed three times for 5 minutes each with fresh cacodylate buffer, followed by three 5-minute washes with 1% BSA in PBS and three 5-minute washes in distilled water. Next, the preparations were incubated with gold enhancement mixture (Nanoprobes, Yaphank, NY) for 8 minutes and then washed with distilled water. Cells were then treated for 2 hours with 1% osmium tetroxide and dehydrated in 50% to 100% alcohol series for three 5-minute changes of each dilution. Cell monolayers were detached with propylene oxide and pelleted for 10 minutes at 18,400 × *g* for 20 minutes at 4°C in an Eppendorf 5254R. Cell pellets were washed twice with propylene oxide, embedded overnight in EMBED 812 1:1 in propylene oxide, contrasted, and analyzed in a Jeol JEM-100 CXII transmission electron microscope.

### Immunoblotting.

HEp-2 cells infected with HRSV were harvested at appropriate times postinfection using 0.05% EDTA, suspended in lysis buffer (50 mM Tris pH 7.5, 150 mM NaCl, 10% [vol/vol] Glicerol, EDTA 5 mM, 1% Triton 100X) for 25 minutes on ice and centrifuged at 18,400 × *g* for 20 minutes at 4°C in an Eppendorf 5254R to separate the nuclear fraction. The supernatant was collected and separated in 10% polyacrylamide gel and transferred onto a nitrocellulose membrane. Subsequently, the nitrocellulose membrane was incubated with the appropriate antibody, namely, goat anti-RSV (ab20745; Abcam), rabbit polyclonal anti-TGN46 (ABT95; Millipore), sheep polyclonal anti-TGN46 (A21448; Thermofisher), rabbit polyclonal anti-SNX1, rabbit polyclonal anti-SNX2, rabbit polyclonal anti-GAPDH (G9595, Sigma), mouse anti-beta actin (sc-47778; Santa Cruz), rabbit polyclonal anti-EGFR (AB52894; Abcam), mouse monoclonal anti-EEA1 (612656; BD Biosciences), and rabbit polyclonal anti-LAMP1 (anti-CD107-A, 555798; BD Biosciences). Except for anti-beta actin antibody, which was incubated for 1 hour, all the others were incubated overnight. This was followed by three 5-minute washes in PBS with 0.1% Tween 20, and then incubation was done with the secondary antibodies, namely, HRP-goat anti-rabbit (656120; Invitrogen), HRP-rabbit anti-goat (305 035 003; Jackson ImmunoResearch), and HRP-rabbit anti-mouse (A9044; Sigma), and then detected with luminol. The images were acquired in a Bio-Rad ChemiDoc.

### Immunoprecipitation.

The protocol used was published by Sugrue et al. (45). Briefly, HEp-2 cells were infected with HRSV at an MOI of 0.1 for 48 hours and then detached with 0.05% EDTA in PBS for 15 minutes. Cells were lysed with lysis buffer followed by clarification and incubation overnight with primary rabbit polyclonal antibody to SNX2 (HPA037400; Sigma-Aldrich) and then incubated with G protein-coupled Sepharose beads (17061801; GE Healthcare) for 3 hours. After three washes in the same buffer, elution was done with beta-mercaptoethanol in sample buffer and subjected to electrophoresis with transference to a nitrocellulose membrane. Immunoblots were done with rabbit anti-SNX2 (SNX2 (HPA037400; Sigma-Aldrich) and goat anti-RSV (ab20745; Abcam), followed by incubation with HRP-goat anti-rabbit (656120; Invitrogen) or HRP-rabbit anti-goat. The images were acquired in a Bio-Rad ChemiDoc.

### Proximity ligation assay (PLA).

HEp-2 cells were grown on coverslips and inoculated with HRSV at an MOI of 1 or mock, and after 24 hours, the cells were fixed with 4% PFA for 15 minutes and permeabilized with 0.01% saponin in PBS. The cells were incubated with a block solution from the PLA kit (DUO82049; Duolink *in situ*) and then with primary antibodies in the following combinations: rabbit polyclonal anti-RSV N and mouse polyclonal anti-RSV M, or rabbit polyclonal anti-SNX2 and mouse polyclonal anti-RSV M. HRSV and mock-inoculated cells were incubated only with mouse anti-HRSV M as biological and technical controls, respectively. After primary antibodies, the cells were incubated with the PLA probes, and the preparation was subjected to the steps proposed by the manufacturers. Lastly, the coverslips were washed five times with the buffer solution provided by the PLA kit, mounted with Fluoromount, and analyzed in a Zeiss laser scanning 780 confocal microscope (Zeiss, Jena, Germany).

### Real-time PCR.

The cells and supernatants from small interfering RNA experiments were collected and treated with TRIzol reagent (Invitrogen, USA) in a proportion of 750 μl of TRIzol to 250 μl of sample. The RNA extraction was done using the TRIzol protocol proposed in the manufacturer’s protocol. Reverse transcription was carried out with the high capacity cDNA reverse transcription kit (Life/Applied Biosystems, USA) using the manufacturer-provided protocol. Briefly, cDNA was obtained from 1 mg of total RNA denatured at 95°C for 5 minutes in the presence of 20 mM primer directed to sequences within the leader-NS1 gene of the HRSV genome. Tubes were kept on ice for 5 minutes while the reactions were assembled with 2 μl of 10 × RT buffer, 0.8 μl of 100 mM dNTP, 1 μl of MultiScribe reverse transcriptase, and water up to the volume of 20 μl. The reaction mixtures were incubated for 10 minutes at 25°C and then for 120 minutes at 37°C, and 5 minutes at 85°C. The PCR mixture contained 5 μl of SYBR green PCR master mix (Kappa, USA), 20 nM forward and reverse primers to the HRSV leader region (forward ACA ACA AAC TTG CGT AAA CCA AAA, reverse CCA TGC TAC TTC ATC ATT GTC AAA CA), 1 μl of specific cDNA, and water to a final volume of 10 μl. Cycling parameters were 50°C for 2 minutes and 95°C for 10 minutes, followed by 45 cycles of 95°C for 30 seconds and 60°C for 1 minute; subsequently, one cycle was done of 95°C for 15 seconds, 60°C for 30 seconds, and 95°C for 15 seconds, and specific amplification was confirmed by analyzing the melting curve. Samples were set up in triplicate on a thermocycler ABI 7500 (Applied Biosystems).

### Small interfering RNA assays for SNX1 and SNX2.

HEp-2 cells were seeded in 12-well plates (containing coverslips) in 40% of confluence; the second siRNA shot (40 nM each siRNA per well) was performed and 48 hours after the first shot, and then the cells were infected with HRSV (MOI = 1). Then, 24 and 48 hours postinfection, the supernatant and cells were harvested for plaque assay, IF, real-time PCR, and Western blot analyses as described above. The sequences of each siRNA were siSNX1 (AUG AAG AAC AAG ACC AAG AGC CCA C) (IDT, Inc., Brazil) and siSNX2 (AAG UCC AUC AUC UCC ACC AAG AGC CAC). The scramble sequence was acquired from Sigma-Aldrich, Brazil.

### Drug treatment.

HEp-2 cells were seeded on coverslips and infected with HRSV (MOI = 1), and 4 hours postinfection, fresh medium was replenished (2% FSB in MEM) containing 10 μM brefeldin A (BFA) in DMSO or DMSO alone. The media containing BFA or DMSO vehicle were maintained for 5 hours, and then BFA or vehicle was washed out, while other wells were maintained to complete 19 hours of drug treatment and 24 hours of infection. Then, after 24 hours of infection, all the cells were fixed with 4% PFA in PBS. After fixation, the cells were subjected to IF as described above. For the experiment reported in Fig. S3, the HEp-2 cells were infected with HRSV at an MOI of 1. Next, 4, 8, 12, and 20 hours postinfection, the cells were incubated with medium (2% FSB in MEM) containing 10 μM BFA. Then, 24 hpi, the cells were fixed with 4% PFA, and stained for HRSV N, giantin, and DAPI.

### Cell fractionation.

This protocol was based on a previous paper by Perez-Victoria (22, 46). HEp-2 cells were infected with HRSV (MOI = 1). After 24 hours, the supernatant from infected and mock-infected cells was discarded, and the monolayers were washed three times with PBS, followed by detaching with PBS with 0.5M EDTA. The cells were subjected to centrifugation at 200 × *g* for 5 minutes; the pellets were washed with PBS and with STE buffer (250 mM sucrose, 20 mM Tris-HCl pH 7.4, 1 mM EDTA, with protease inhibitors) to produce an osmotic shock. Cells were passed 20 times through a 25G needle for cell lysis in 0.5 ml of STE buffer without sucrose. The cell homogenates were centrifuged at 1,000 × *g* for 2 minutes to remove nuclear contents, and the post-nuclear supernatants were loaded on top of a discontinuous 10 to 30% (wt/vol) glycerol gradient in STE buffer, laid on 0.5 ml of 80% sucrose cushion in ultracentrifuge tubes. After that, tubes were centrifuged at 29,000 rpm for 2 hours in a Thermo TH629 rotor. After centrifugations, fractions (1.250 ml) were carefully collected from top to bottom of the ultracentrifuge tubes. Then, trichloroacetic acid (TCA) was added to each collected fraction for protein concentration, and the pellet was suspended in sample buffer with 4% beta-mercaptoethanol. The samples were heated at 95°C, loaded in 10% SDS-PAGE gel, transferred to nitrocellulose membrane, and analyzed by Western blotting.
