# Supplementary figures and images for: Host Retromer Protein Sorting Nexin 2 Interacts with Human Respiratory Syncytial Virus Structural Proteins and is Required for Efficient Viral Production
Source: mBio. 2020 Sep 29;11(5):e01869-20. doi: 10.1128/mBio.01869-20 (PMC7527724; doi:10.1128/mBio.01869-20)

Supplementary Figure 1.

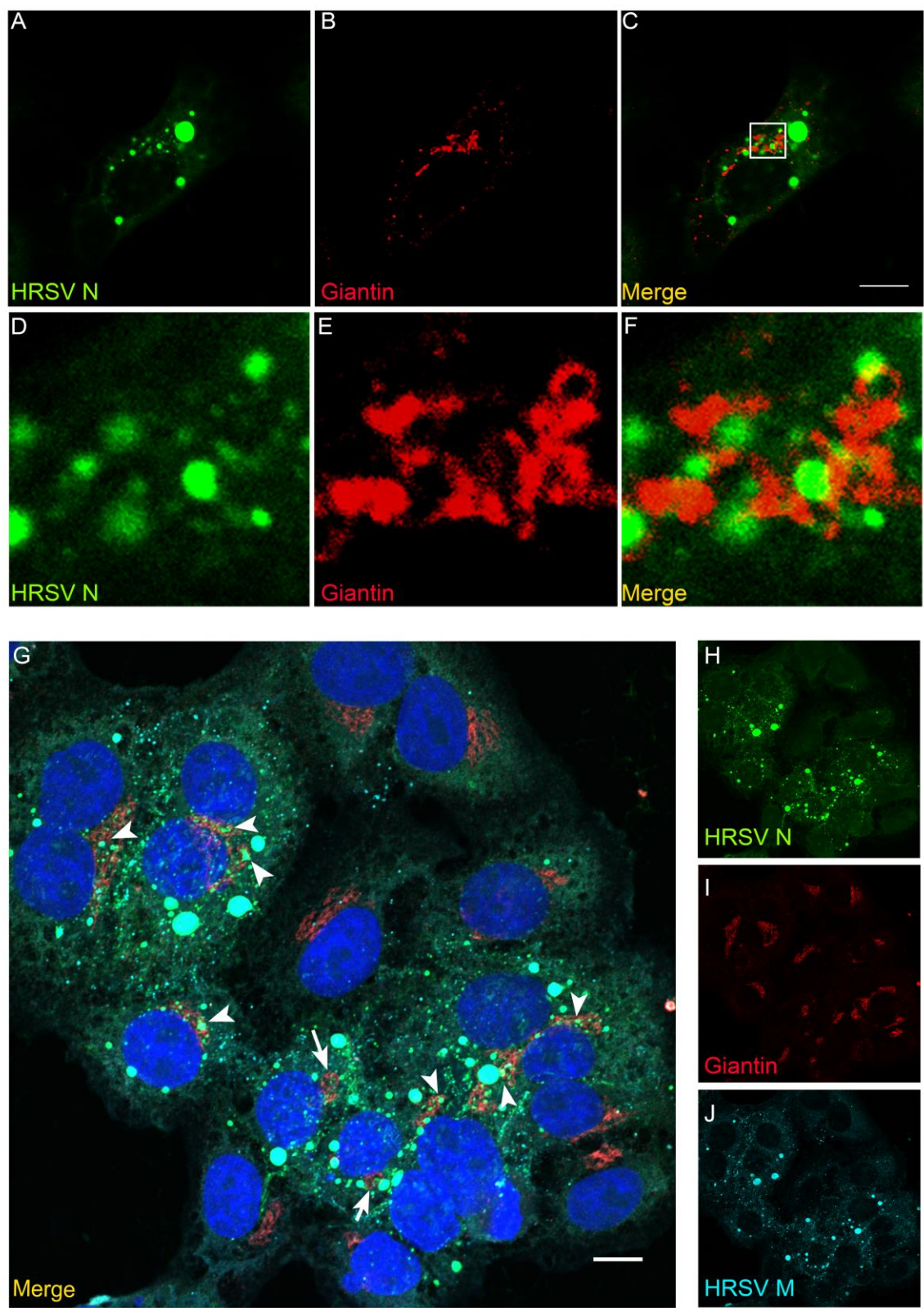

Supplement: FIG S1 [file mBio.01869-20-sf001.pdf]

Supplementary Figure 2.

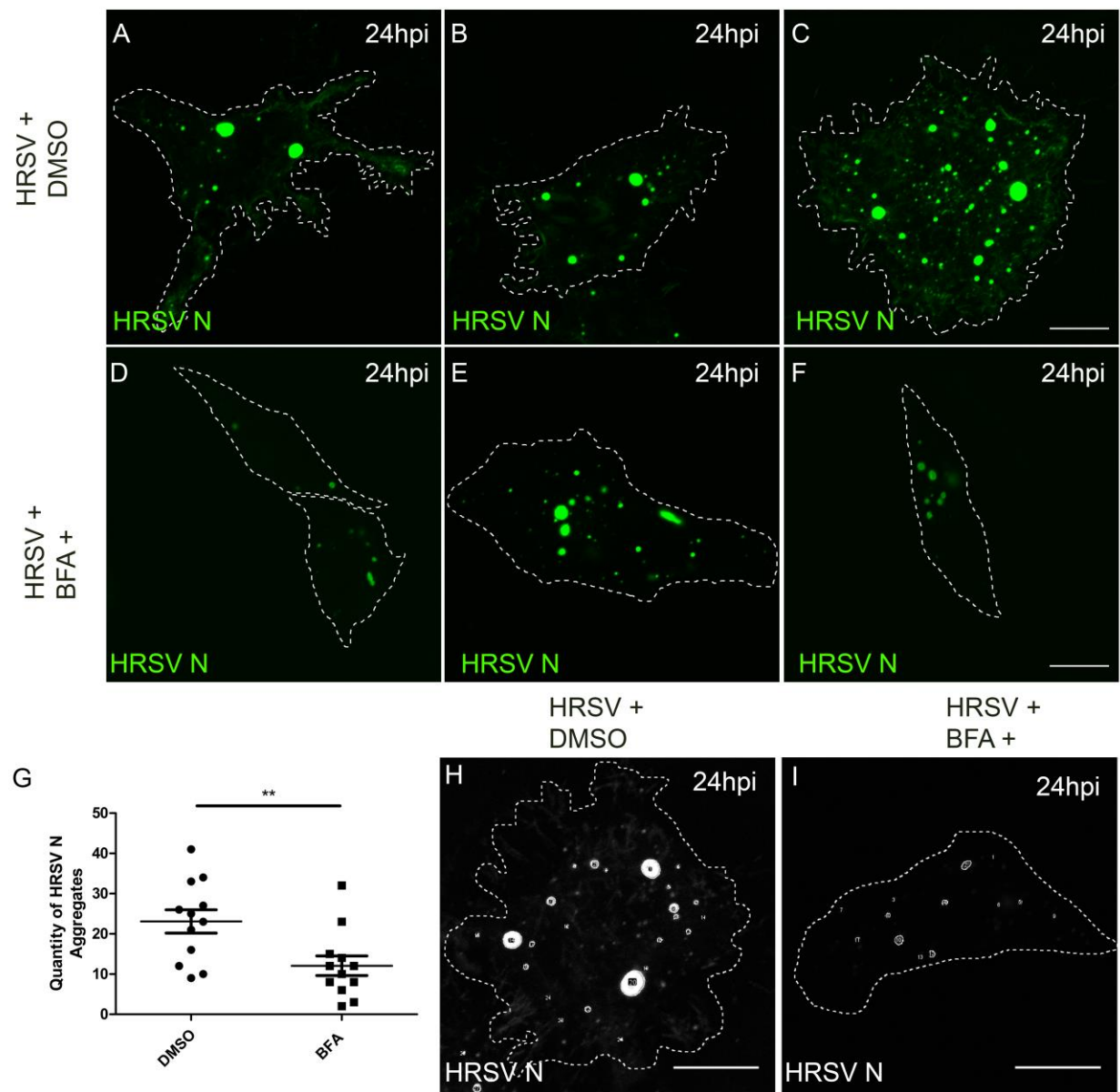

Supplement: FIG S2 [file mBio.01869-20-sf002.pdf]

Supplementary Figure 3.

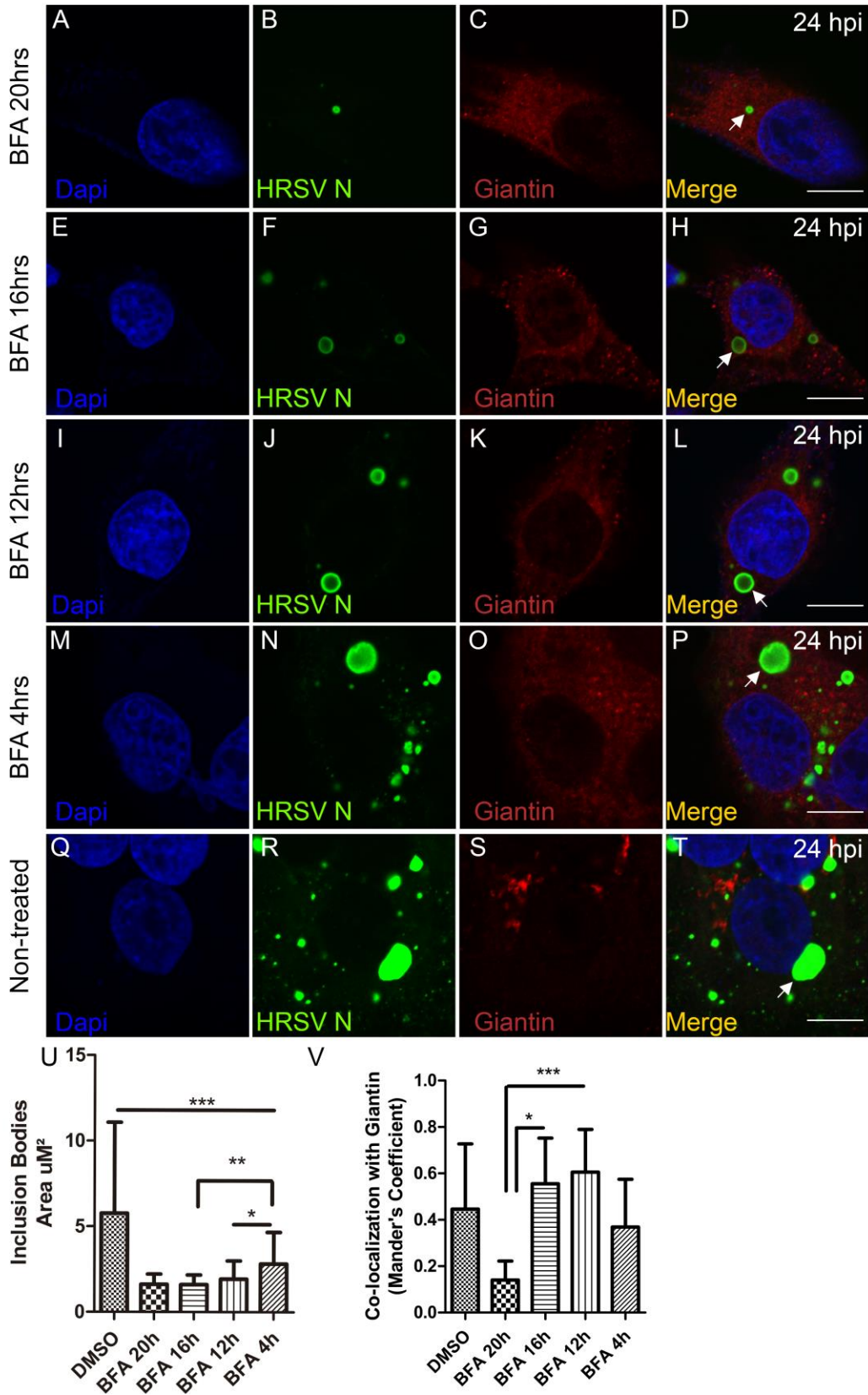

Supplement: FIG S3 [file mBio.01869-20-sf003.pdf]

Supplementary Figure 4.

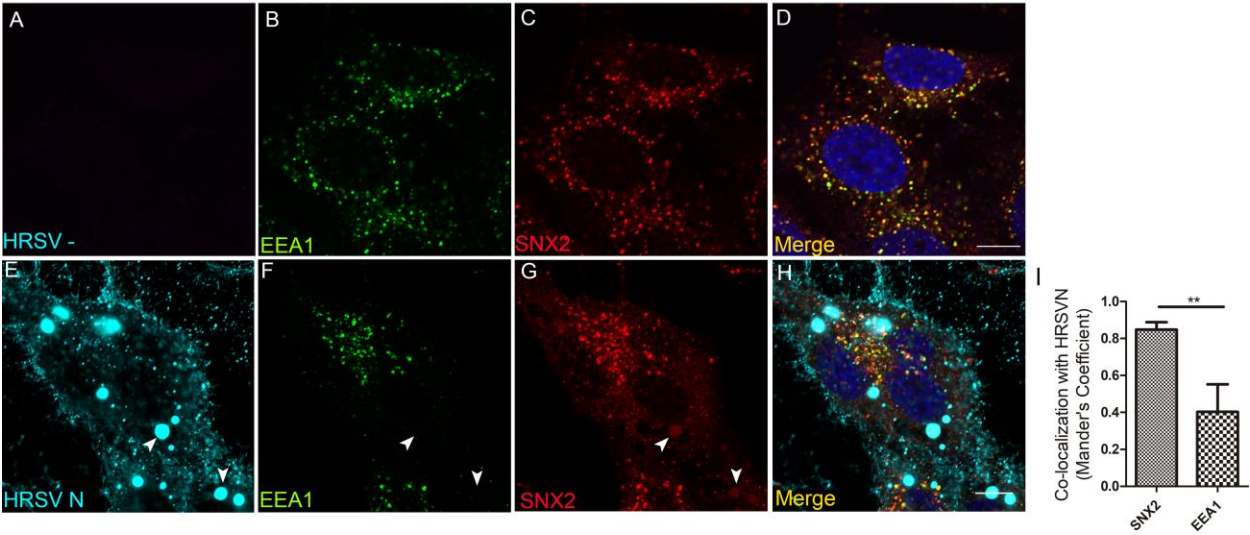

Supplement: FIG S4 [file mBio.01869-20-sf004.pdf]
